
JOURNAL INFORMATION
==============================
NLM Title Abbreviation: J Orthop Traumatol
Journal ID: J Orthop Traumatol
NLM Journal ID: 101090931

Journal of Orthopaedics and Traumatology : Official Journal of the Italian Society of Orthopaedics and Traumatology

ISSN: 1590-9921
EISSN: 1590-9999
Publisher: Springer

ARTICLE INFORMATION
==============================
PMCID: PMC4417756
DOI: 10.1007/s10195-012-0210-2
Article ID: 210
Article version: 1
Subjects: Events

In-Depth Oral Presentations and Oral Communications

Electronic publication date: 2012 Aug 30
Print publication date: 2012 Nov
Volume: 13
Issue: Suppl 1
First page: 57
Last page: 89
Copyright: © The Author(s) 2012
License: Open AccessThis article is distributed under the terms of the Creative Commons Attribution 2.0 International License (https://creativecommons.org/licenses/by/2.0), which permits unrestricted use, distribution, and reproduction in any medium, provided the original work is properly cited.
License URL: https://creativecommons.org/licenses/by/2.0/

Keywords: Anterior Cruciate Ligament, Total Knee Arthroplasty, High Tibial Osteotomy, Dabigatran Etexilate, Plantar Fascia
issue-copyright-statement: © The Author(s) 2012

==============================
J Orthopaed Traumatol (2012) 13 (Suppl 1):S57–S89
DOI 10.1007/s10195-012-0210-2

12 NOVEMBER 2012

### In-Depth Oral Presentations and Oral Communications

### IN-DEPTH ORAL PRESENTATIONS

*AT05–HAND AND WRIST*

### Treatment of malunion of the proximal phalangeal fractures of the hand

V. Potenza*, S. Bisicchia, R. Caterini, A. Fichera, P. Farsetti, E. Ippolito

Università di Roma Tor Vergata (Rome, IT)

**Introduction** It is difficult to treat fractures of the phalanges of the hand because they can cause complications such as deformity and joint limitation with a reduction in the grasping function. The most frequent complications are malunion of the fracture and joint limitation. The greatest incidence of complications can be found in transverse fractures of the base of the proximal phalanx, in articular fractures, comminuted fractures, and in those associated with lesions of the soft tissues.
**Materials and methods** In this paper, we describe the clinical and radiographic results obtained in 20 patients (for a total of 24 fingers) treated surgically to correct the malunion of a fracture of the proximal phalanx of the hand. The patients (16 male and 4 female) were operated on at a mean of 40 days after the initial treatment. A corrective osteoclasia was done within 25 days after initial treatment in 10 cases. In the other 14 cases we performed a corrective osteotomy. Patients were followed-up clinically and radiologically evaluating the deformity correction, the range of motion and grip strength of the hand, and the patient's degree of satisfaction. We also evaluated functional results by DASH (Disabilities of the Arm, Shoulder and Hand) questionnaire.
**Results** At a mean follow-up of 24 months after surgery, we obtained a correction of the deformity in all patients. An improvement of about 30 % in the range of motion of the PIP was observed. Four patients complained about pain at the maximum degrees of articular excursion of the MP and PIP joints. All the patients presented an improvement in the grip strength of the hand. The mean DASH score in our series was 5 points. X-rays showed that all osteoclasia-osteotomies performed healed within 8 weeks. In 4 cases of articular incongruency, X-rays showed no signs of osteoarthritis in the PIP joint.
**Discussion** The main cause of complications of phalangeal fractures of the hand is the choice of an unsuitable treatment, subsequent to a mistaken clinical and radiographic assessment of the factors that can influence the result of treatment, such as localization, geometry, and stability of the fracture and the presence of associated soft tissue lesions.
**Conclusions** Immediate surgical treatment of articular and peri-articular unstable fractures of the proximal phalanx is advised to achieve a stable synthesis and an early mobilization of the MP and IP joints. However, if a malunion is present, it has to be corrected surgically as soon as possible.

### Radio-distal epiphysis fractures: treatment with angular stability plate of latest generation

R. Di Virgilio*, E. Coppari, E. Condarelli, M. Rendine

(Rome, IT)

**Introduction** Distal radius fractures are the most common fractures of the upper limb and coincide with 17 % of all fractures treated in emergency rooms. The incidence of these fractures is greater in patients aged 6 to 10 years, and in those between 60 and 70 years. In older patients the incidence is higher in females. In the articular fractures, displaced, dislocated and highly unstable is indicated open internal fixation (ORIF) to restore the congruity of the joint surface, to restore the correct length of the radius, its inclination and palmar tilt. Moreover, the volar approach is less invasive, respect tendons, allows, if it is intact, the cover plate by the pronator quadratus muscle, and can also be used to treat dorsal fractures.
**Materials and methods** Many authors have proposed different classifications, but we preferred to use the AO classification that focuses on the increasing severity of bone injury. The purpose of this study was to evaluate the efficiency of the latest generation volar plate in the treatment of articular fractures of the wrist type B and C of the AO classification. From January 2005 to December 2011, 214 distal radius fractures were surgically treated with ORIF volar approach and plates with angular stability. The follow-up was on average 3 months, all patients were remotely controlled by evaluating the bone consolidation, ROM, grip strength, clinical and functional results according to the Mayo Modified Wrist Score Card and the modified DASH questionnaire.
**Results** All patients had consolidation of the fracture and using as parameters the questionnaire and the Dash board MWS modified, the authors obtained 90 % excellent and good results. 10 % between discrete and bad, with an almost disappearance of pain and in some cases a flexion limitation of approximately 10° and/or pronation-supination limitation.
**Discussion** In consideration of the reduced recovery time, recovery of full or slightly restricted range of motion, patient satisfaction and low complication rates, we believe that is the preferred method of choice in this type of fracture (AO, B and C).
**Conclusions** This retrospective work shows how the internal fixation with plate and screws with angular stability through volar approach is particularly recommended for unstable fractures of the distal radius, it allows early mobilization, a complete recovery of function and a *restitutio ad integrum* of the affected joint an important decrease of post-traumatic arthrosis of the radio-carpal joint.

### *AT06–SPINE*

### Arm Squeeze Test: a new clinical test for differential diagnosis between cervical nerve root compression and shoulder disease

S. Gumina, S. Carbone, V. Arceri*, P. Albino

Ortopedia e Traumatologia, Università di Roma Sapienza (Rome, IT)

**Introduction** Objective of this study is to evaluate the diagnostic values of the Arm Squeeze Test. The test consists in squeezing the middle third of the upper arm. Our hypothesis is that squeezing is responsible for pain only in patients with cervical nerve root compression from C5 to T1.
**Materials and methods** 1567 patients were included in this study. Diagnosis of cervical nerve root compression or shoulder disease was clinically and strumentally formulated before performing test. Secondary, patients were subdivided as follow: 903 with rotator cuff tear, 155 with shoulder adhesive capsulitis 101 with acromioclavicular (AC) joint arthropathy, 55 with calcifying tendonitis, and 48 affected by gleno-humeral arthritis. The study sample included 305 patients with cervical nerve root compression from C5 to T1 with pain irradiating to one or both shoulders. 350 healthy volunteers were recruited as controls. The test was positive when score on a VAS Scale was 3 points or higher on squeezing the middle third of the upper arm compared to AC joint and antero-lateral-subacromial area. Results were analysed with Pearson $\chi^2$ Test.
**Results** The test was positive in 295/305 (96.7 %) of patients with cervical nerve root compression, compared to 35/903 (3.87 %), 3/155 (1.93 %), 0/101 (0 %), 1/55 (1.81 %), 4/48 (8.33 %) of those with rotator cuff tear, adhesive capsulitis, AC arthropaty, calcifying tendonitis and glenohumeral arthritis, respectively. A positive result was obtained in 14/350 asymptomatic subjects (4 %). If cervical nerve root compression was compared to shoulder conditions, the test had sensitivity from 0.97 to 0.99 and specificity from 0.93 to 0.96. Respect to controls, sensitivity was 0.98 and specificity was 0.97.
**Discussion** Differential diagnosis between cervico-brachialgy and shoulder pain is often challenging.
**Conclusions** The Arm Squeeze Test may be useful to distinguish cervical nerve root compression from shoulder disease in case of doubtful diagnosis.

### Posterior only fusion with pedicle screws in the treatment of Scheuermann kyphosis

M. Di Silvestre*[1], F. Lolli[1], G. Bakaloudis[2], F. Vommaro[1], K. Martikos[1], A. Ortolani[1], A. Baioni[1]

[1]Chirurgia delle Deformità del Rachide, Istituto Ortopedico Rizzoli (Bologna, IT);
[2]San Carlo di Nancy (Rome, IT)

**Introduction** The surgical treatment of Scheuermann kyphosis in the past generally consisted of a combined (anterior and posterior) fusion. Recently, only posterior correction is more often performed. Aim of the study is to evaluate results of posterior only approach in the treatment of Scheuermann kyphosis.
**Materials and methods** Twenty-one consecutive patients (8 males, 13 females) were included in the study. The patients were treated by posterior arthrodesis with pedicle screws and Ponte osteotomies at all levels (10.1 levels on average). The mean age was 19.9 years and the "maximum kyphosis" 89.6° (range, 72.1°–105.2°).
**Results** All 29 patients were reviewed at a minimum follow-up of 2 years (maximum of 3 years and 9 months). The average operating time was 279 min with an average blood loss of 795 cc. The correction of maximum kyphosis was of 63.7 % (range 52–71 %). There were no neurological complications: two SSEP/PEM reductions resolved intraoperatively without clinical sequelae. There was no loss of correction at follow-up. The complications were represented by 2 proximal junctional kyphoses (9.5 % of cases) (1 of which required the extension of the synthesis of 1 level proximally) and 1 loosening (4.7 %) of a distal screw (treated with distal extension of the instrumentation by 1 level).
**Discussion** Posterior only approach ensured satisfying kyphosis correction with a low complications incidence.
**Conclusions** Posterior correction alone represents an effective method in the treatment of Scheuermann kyphosis.

### Cervical disc arthroplasty versus fusion: a systematic literature review

A. Di Martino, R. Papalia, B. Zampogna, G. Salvatore, N. Papapietro, V. Denaro

Department of Orthopaedics and Trauma Surgery, University Campus Bio-Medico of Rome (Rome, IT)

**Introduction** Cervical disc arthroplasty and anterior cervical discectomy and fusion are nowadays considered as the main surgical alternatives for the treatment of cervical disc herniation. The aim of this systematic review is to evaluate which surgical procedure between these two gives better clinical and radiological results, and has lower failure rates and surgical-related complications.
**Materials and methods** A research in literature has been led using PubMed, Cochrane and Google Scholar, setting as endpoint the 25th of February and inserting the following keywords: Cervical spine arthroplasty, cervical spine replacement, cervical disc prosthesis versus anterior cervical discectomy and fusion. We identified 56 articles published in indexed peer reviewed journals; among these we included in this systematic review only I and II level evidence studies.
**Results** We identified 13 clinical studies, including about 3,000 patients, with a mean age of 44 years. Several kind of implants and surgical techniques of segmental fusion have been employed to treat cervical disc herniation. We obtained good clinical results comparing clinical scores, ROM and radiographic evaluations before and after surgery, despite in some studies we noticed several differences considering failure rates, relapse and quality life index of patients.
**Conclusions** Given the relatively short follow-up time, the results of the two different techniques should be evaluated comprehending studies with a longer follow-up (> 36 months), in order to establish statistically significant differences and the primacy of one of the two techniques in the treatment of patients affected by cervical disc herniation.

### *AT07–BASIC SCIENCE*

### Osteogenesis from adipose tissue: an in vitro study

A. Pozzuoli*[1], M. Zamperetti[1], B. Zavan[2], M. Inferrera[2], A. Gasparella[1], R. Aldegheri[1]

[1]Clinica Ortopedica, Università di Padova (Padua, IT);
[2]Dipartimento di Scienze Biomediche, Università di Padova (Padua, IT)

**Introduction** The growing interest towards adipose tissue as source of mesenchymal stem cells for bone tissue engineering and the introduction of bone demineralized matrices (DBM) able to stimulate the regeneration of fully functioning bone tissue represent new tools available to the orthopaedic surgeon for the treatment of wide bone defects. The aim of this study was to evaluate the ability of DBM to stimulate in vitro the proliferation and the osteogenic differentiation of mesenchymal stem cells isolated from adipose tissue (hASCs). Moreover, the safety of the method was investigated using molecular genetic tests.

**Materials and methods** hASCs were isolated, by enzimatic digestion, from lipoaspirate and morphologically characterized using immunofluorescence's techniques. Then, hASCs were seeded on two osteoinductive matrices consisting of human demineralized bone, in presence of non osteogenic medium, and tested for cell proliferation by vitality assay. Osteogenic differentiation of hASCs was evaluated, by real-time RT-PCR, analyzing gene expression of the following markers: RUNX2, osteocalcin, osteonectin, type I collagen, BMP2, and PPARgamma. Any chromosomal aberration was studied with CGH-array.

**Results** Cells isolated and amplified on monostrate, express stem markers. Cell proliferation on decellularized matrix is positive and increasing over time, with the same trend also observed in the analysis of gene expression of osteogenic markers like RUNX2, osteocalcin, osteopontin, type I collagen, and BMP2. Gene expression of adipogenic transcriptional factors like PPARgamma is very low. None chromosome abnormalities are present in all the cultures at every passages.

**Discussion** Positive stem markers confirm the isolation of stem cells from adipose tissue. The vitality test shows that cells proliferate and amplify in presence of DBM, confirming its biocompatibility. Positive osteogenic markers indicate that ASCs acquire osteogenic phenotype in presence of DBM. Finally, absence of chromosomal alterations guarantees the safety of biomaterial and of ASCs.

**Conclusions** The results show that hASCs adhere, proliferate, and differentiate in osteoblast-like cells, if seeded in presence of DBM in non osteogenic medium, confirming the osteoinductive capacity of the biomaterial. Moreover, chromosomal analyses confirm the safety of cultures and biomaterial. Therefore, hASCs can be considered a promising source of osteoprogenitor cells for bone regeneration.

### Medial femoral fractures associated with chronic kidney disease (CKD): a structural investigation of the subchondral bone by histomorphometry study

P. Pistillo*, E. Gasbarra, R. Iundusi, J. Baldi, F. Perrone, L. Paganizza, S. Santini, U. Tarantino

Università degli Studi di Roma "Tor Vergata" (Rome, IT)

**Introduction** Chronic Kidney Disease (CKD) is associated with an increased risk of fragility fractures. Our intent is to analyze, through histomorphometric study, morphological and structural changes caused by mild to moderate CKD in trabecular bone.

**Materials and methods** After informed consent, we enrolled 70 osteoporotic patients over-55 (men and women), with medial femoral fracture, who underwent hip replacement surgery. Patients were divided into two groups: one composed of 35 subjects with mild to moderate CKD (Group A), and a control group of 35 subjects with normal kidney function (Group B). The diagnosis of osteoporosis was made by DEXA examination (GE Lunar DXA-i) while the evaluation of bone metabolism was made by determination of serum markers of bone turnover. The Stadium of CKD was determined by calculating the volume of glomerular filtration rate (GFR). Histomorphometric evaluations (Bioquant Osteo, BIOQUANT Image Analysis Corporation) were performed using a lamina of the femoral head taken at the end of surgical procedure. We measured: Bone Volume (BV), Bone Surface (BS), Trabecular Thickness (Tb.Th.) and Trabecular Separation (Tb.Sp.).

**Results** Comparing the two groups of patients, histomorphometric study revealed significance for the following values: BV (Group A $4.13 \pm 1.23$ mm$^2$ vs. Group B $92.68 \pm 19.47$ mm$^2$, $p < 0.0001$) and Tb.Th. ($86.13 \pm 21.16$ uM Group A vs. Group B $115.35 \pm 8.50$ uM; $p < 0.001$) reduced in the group of patients with CKD compared with control group; Tb.Sp. (Group A $280.74 \pm 82.28$ uM vs. Group B $192.91 \pm 70.05$ uM $p < 0.001$) increased in the group of patients with CKD compared to the control group. Patients affected by CKD (Group A) showed values of ALP (Group A $79.92 \pm 3.95$ IU/L vs. Group B $61.78 \pm 17.75$ IU/L, $p < 0.05$), PTH (Group A $103 \pm 12.5$ pg/ml vs. Group B $72.4 \pm 10.8$ pg/ml, $p < 0.001$) and phosphorus (Group A $5 \pm 0.8$ mg/dl vs. Group B $3.9 \pm 0.9$ mg/dl, $p < 0.05$) higher than control group.

**Discussion** In patients with mild to moderate CKD there are both metabolic and morpho-structural bone changes confirming the correlation between kidney function and skeletal disorders.

**Conclusions** Patients with mild to moderate CKD show an early loss of bone tissue. It is therefore necessary to perform an accurate assessment of biochemical, morphological and densitometric parameters in order to set an effective therapy to reduce the risk of fragility fractures in CKD of elderly patients.

### Physiological changes of the knee laxity during growth

F. Falciglia, A. Poggiaroni*, M. Giordano, A.G. Aulisa, V. Guzzanti

Ospedale Pediatrico Bambino Gesù (Rome, IT)

**Introduction** With increasing loss of flexibility and laxity, there is a change in tolerable physiological and biomechanical forces to intra-articular structures. Joint movement can decrease because of increased musculotendinous tightness and reduction in elasticity of the ligaments. If an imbalance in flexibility is created, increased functional overload is more likely to occur. The current study attempts to elucidate the relationship between flexibility and laxity in relation to age, gender, Tanner stage, and anthropometric measures to avoid functional overloads in adolescents.

**Materials and methods** A two-phase (cross-sectional and longitudinal) study assessed knee joint laxity and flexibility in 172 normal non-athletic adolescents (10.5–14.5 years), using a KT 2000 arthrometer, anthropometric measurements, and Carter and Wilkinson tests. Clinical anthropometric values recorded were weight, height, length of the lower limbs, and thigh diameter. Correlation of these evaluations was done with gender and Tanner stage. Subjects were serially evaluated over a two-and-one-half year period, with a minimum of three and a maximum of five observations at planned intervals of 6 months for a total of 553 measurements. Statistical analysis was performed to point out correlations between the various parameters and significant differences from left and right side.

**Results** Increased flexibility was seen significantly more frequently in females than males in both study phases. Age, Tanner stage, and anthropometric values were not significantly associated with laxity in the cross-sectional study. In the longitudinal study, an inverse relationship was demonstrated between Tanner stage and KT 2000 laxity measures after adjusting for other variables. Sequential evaluation showed a progressive decrease of sagittal laxity at the onset of Tanner stage 2. Laxity was significantly greater in adolescents, with signs of joint physiologic hyperflexibility.

**Discussion** Our longitudinal data confirm a progressive reduction in sagittal laxity during the rapid, peak height velocity of pubertal growth. Physiological laxity measures must be considered in relation to growth. In particular it is expected that the initial change in laxity occurs in Tanner stage 2–3.
**Conclusions** Evaluation of laxity and flexibility during the adolescent growth phase is important for a better definition of muscle strengthening or flexibility programs, to avoid functional overloads and injury in adolescents.

### In vitro stimulation of primary human tendon stem progenitor cells (hTSPCs) by pulsed electromagnetic fields (PEMFs)

D. Stanco*[1], L. De Girolamo[1], P. Romeo[2], S. Setti[3], M. Viganò[1], A. Lovati[4], G. Thiebat[1], V. Sansone[2]

[1]IRCCS Istituto Ortopedico Galeazzi (Milan, IT);
[2]Clinica Ortopedica, Università degli Studi di Milano, IRCCS Istituto Ortopedico Galeazzi (Milan, IT);
[3]IGEA s.p.a (Carpi, IT);
[4]Gruppo Ospedaliero San Donato Foundation (Milan, IT)

**Introduction** Sports-related tendon injuries are common and functional recovery is often problematic. Pulsed electromagnetic fields (PEMFs) can be effective in the management of musculoskeletal tissue healing, but the biological mechanism of action remains partially unexplained. In this study we investigated the effects of PEMFs on primary human tendon stem progenitor cells (hTSPCs).
**Materials and methods** hTSPCs were isolated from semitendinosus tendon of 10 healthy donors (31 ± 5 years old) that underwent to ACL reconstruction. After having analyzed the immunophenotype profile of hTSPCs by cytofluorimetric analysis, cells were exposed to a single PEMFs exposure (1.5 mT, 75 Hz) (4, 8 or 12 h) or daily treatments of 8 and 12 h for 4 days. Cells viability and proliferation were assessed by MTT assay and CyQuant® assay kit respectively after 0, 2, 7 and 10 days. Moreover, we assessed the specific modulation of gene expression like COL1A1, SCX and TNMD in untreated (CTRL) and treated cells.
**Results** The mean yield of hTSPCs after isolation was 4.4 ± 7.9 × 105 cells per grams of digested tendon tissue. hTSPCs exposed to PEMFs presented the same fibroblast-like morphology as CTRL cells. The single 12 h-PEMFs exposure induced an increase of cells viability respect to untreated cells (+14 %) that was maintaining until 10 days. hTSPCs treated for 8 h showed, at day 0, a significant increase of DNA content in comparison to untreated cells (+25 %, $p < 0.05$) and similar effect was observed after 12 h-exposure at 2 and 7 days (+16 and +8 %, respectively). Multiple PEMFs treatments seem to have comparable effects on cells viability and DNA content respect to single-exposed cells. Tissue specific gene expression COL1A1, SCX and TNMD was differently affected by the different PEMFs treatment.
**Discussion** Our findings show that PEMFs stimulation affects cells viability, suggesting the cytocompatibility of the treatment. Moreover, 8 and 12 h PEMFs exposure are able to enhance cell proliferation respect to CTRL cells; these results, together with a modulation of tendon specific gene expression, could explain the beneficial effect of PEMFs in tendon healing observed in the clinical practice. Further experiments are in progress to better comprehend the biological response of tendon cells to PEMFs.
**Conclusions** Our in vitro study suggest that PEMFs exposure are able to affect in vitro hTSPCs viability, proliferation and gene expression; therefore, PEMFs treatment could be a valid clinical approach for early recovery after tendon injuries.

### A model of interaction between muscle and bone: osteoporosis-related sarcopenia

U. Tarantino*, M. Celi, J. Baldi, R. Iundusi, D. Lecce, F.M. Liuni, G. Fioravanti Cinci, E. Gasbarra

Università degli Studi di Roma "Tor Vergata" (Rome, IT)

**Introduction** Osteoporosis and sarcopenia are two age-related diseases which cause disability. Aim of the study was to evaluate the degree of fibres atrophy in the vastus lateralis muscle of patients with osteoporosis and to define the role of the signalling pathway IGF-1/PI(3)/Akt in the pathogenesis of osteoporosis-related sarcopenia.
**Materials and methods** After informed consent, we enrolled 60 female patients (age 71.53 ± 9.74) undergoing hip replacement surgery for medial femoral fracture (30 patients, Group A) or osteoarthritis without significant functional limitation (30 patients, Group B). We performed a biopsy of the vastus lateralis muscle fibres in all patients. These muscle fibers, after ATPase staining, were analyzed by optical microscopy, measured and classified. To assess the possible involvement of Akt in determining a particular form of osteoporosis-related muscular atrophy, we selected 12 patients in Group A with greater degree of type II fibers atrophy and 12 patients in Group B used as controls for the immunoblotting study.
**Results** In both groups, type II fiber-atrophy was significantly more frequent than type I fiber ($p$ value < 0.0137 % Group A, Group B 12 %), with a threefold ratio in Group A and only a 1.5-fold in Group B. Type II fiber-atrophy in Group A was related to the osteoporosis degree ($p < 0.05$); in Group B fiber atrophy was related with the severity of pain and the resulting degree of joint limitation. In osteoporotic patients the Akt average was 2.5-fold lower (60 %, $p < 0.01$) compared to that measured in the muscle of Group B.
**Discussion** Patients with osteoporosis had an higher degree of muscular atrophy affecting mainly type II muscle fibers; this seems to be related with the degree of osteoporosis. On the opposite arthritis seems to be related with the severity of pain and the resulting joint limitation. The reduction muscular Akt in osteoporotic patients indicates the involvement of the IGF-1/PI(3)K/Akt signaling pathway in the pathogenesis of osteoporosis-related muscle atrophy.
**Conclusions** Osteoporosis-related muscle atrophy is a systemic disorder which demonstrates the strict correlation between bone and muscle; for this reason we need to develop new therapeutic strategies to improve both bone quality and muscular trophism.

### *AT08–KNEE*

### VISIONAIRE accuracy in knee prostheses: navigator evaluation

R. Iorio, D. Mazza*, G. Bolle, A. Redler, L. Caperna, F. Conteduca, A. Ferretti

Ospedale Sant'Andrea (Rome, IT)

**Introduction** The aim of this study is to evaluate the accuracy and reliability of VISIONAIRE (Smith & Nephew, Inc, Memphis, Tenn) Patient Matched Cutting jigs in both plane and for both components and to compare it with an extra-medullar tibial instrumentation, by analysing data as detected by intra-operative use of VectorVision knee navigation software from BrainLAB (Redwood City, Calif).
**Materials and methods** 12 patients entered the study. Preoperatively all patient underwent a full-length weight-bearing radiograph in A-P

and an MRI according to the protocol. All patients were operated with cemented posterior stabilized prosthesis cruciate ligament sacrificing (Journey BCS, Smith & Nephew, Inc, Memphis, Tenn) by the same surgeon using the VISIONAIRE patient matched cutting jigs. During surgery, once the extra-medullar guides were placed and fixed on the tibia, the orientation on coronal and sagittal plane was checked by the navigator and then he was compared with the data obtained by measuring the orientation of VISIONAIRE Patient Matched Cutting tibial jigs. Then the orientation of the femoral cutting jigs was recorded. An unsatisfactory result was considered an error = 2° in both coronal and sagittal plane for tibial component as a possibible error of 4° could result in aggregate.

**Results** On the coronal plane the mean deviation of the EM tibial guides from the ideal alignment (0°) was 0,7° ± 0,39° and of the VISIONAIRE was 1,29° ± 1,55° ($p = 0{,}22$). On the sagittal plane the mean deviation of the EM tibial guides from 3° of posterior slope was −1,62° ± 1,78° and of the VISIONAIRE was +1,16° ± 4,29° ($p < 0{,}05$). On the coronal plane the mean deviation of the femoral guide from the ideal alignment was 1.2° ± 0.6° and in the sagittal was 3.7° ± 2°.

**Discussion** This preliminary study documented a only fair accuracy of the method with a consistent risk of error of more of 3° especially in the sagittal plane. We could speculate that the problem on the sagittal plane depends on the fact that the preoperative protocol does not include a lateral X-ray projection of the knee and only include AP standing X-rays of the straight-leg and MRI.

**Conclusions** The standard instrumentation in the hands of experienced surgeon could lead to a better alignment of the prosthesis as compared with an MRI based Patient Matched Cutting Jigs.

### Relationship between the posterior offset of femoral condyles and sagittal tibial slope: influence on knee flexion in TKA

P. Sessa, A. Della Rocca*, F.R. Ripani, F. Gabriele, C. Curri, G. Cinotti

Clinica Ortopedica, Università La Sapienza (Rome, IT)

**Introduction** The influence of posterior offset of femoral condyles (POFC) and sagittal tibial slopes (STS) on knee motion in flexion after total knee arthroplasty (TKA) remains controversial. In the present study we assessed the variability of both the POCF and STS and evaluated if any relationship between the two exists which could influence knee flexion after TKA.

**Materials and methods** MRI of the knees of 80 patients with mild to moderate knee pain but with no evidence of degenerative or post-traumatic changes in the joint were assessed. On each MRI, the sagittal longitudinal tibial axis was identified and the STS measured in both the medial and lateral compartments. The POCF was then measured in the medial and lateral condyles with respect to a line tangent to the posterior femoral cortex. Measurements were performed by two examiners using a high resolution imaging software.

**Results** There were 45 men and 35 women with a mean age of 38.9. STS averaged 8° and 7.7° in the medial and lateral side, respectively. The mean POCF was 27.4 mm and 25.2 mm in the medial and lateral condyles, respectively ($p = 0.0001$). The variation coefficient (COV) was 11.5 and 38 % for measurements of POCF and STS, respectively. In the medial compartment, a significant correlation was found between POCF and STS, in the sense that the greater the POCF the larger the STS, and vice versa.

**Conclusions** The results of the present study show that a larger variability exists in the slope of the tibial plateaus than POFC. A significant correlation between the two was found in the medial side, meaning that in order to achieve a proper range of motion and ligamentous balancing in flexion, a mutual relationship should exist between the anatomical structures of the posterior portion of the knee joint.

### Surface cementing technique of tibial component in rotating-platform total knee arthroplasty: is it a good idea?

M. Bruzzone*[1], R. Rossi[1], A. Ferro[1], F. Dettoni[1], A. Marmotti[1], D.E. Bonasia[2], F. Castoldi[1]

[1]Clinica Universitaria, Ospedale Mauriziano Umberto I (Turin, IT); [2]Clinica Universitaria, Ospedale C.T.O. (Turin, IT)

**Introduction** Controversy still exists regarding which cementation technique of the tibial component in total knee arthroplasty (TKA) is preferable. Full cementation (FC) showed excellent long-term outcomes and surface cementation (SC) with fixed-bearing designs provided excellent mid-term results. Concerns have been expressed about possible rotary forces to the tibial rotating platform (RP), when the tibial stem remains cementless, with the risk of early loosening. Recent biomechanical studies showed that SC reduced stress-shielding effect and proximal bone resorption around the tibial component, potentially increasing implant survivorship compared to FC. The starting hypothesis of this clinical study was that, using SC on RP-TKAs, the rate of early loosening and radiolucency lines around the tibial tray would have been comparable to other designs and fixation techniques.

**Materials and methods** We analysed 94 consecutive RP-TKA performed using SC in order to identify any possible correlation between early loosening or radiolucency lines and relevant clinical (BMI, sex, age, follow-up time, clinical scores) of radiographycal (cement penetration, component positioning and alignment) covariates to determine the risk factors for these conditions.

**Results** At a mean follow-up of 46 months, medium Knee Society Score was 168 (range: 162–173), mean cement penetration was 2.5 mm. We had no cases of early implant loosening; in 14 asymptomatic patients (13 %) a radiolucency line was detected around the tibial peg. The presence of radiolucency lines was significantly associated only with age > 75 years.

**Discussion** The present study is the first to report short- to medium-term clinical and radiological results of a unidirectional rotating platform prosthesis using a SC technique and a press-fit stem fixation. The overall results reported are comparable to outcomes reported in literature using FC technique on mobile bearing or SC in fixed platform TKAs. According to the longer follow-up outcomes reported in current literature, FC still remains the gold standard. Nevertheless, along with the recent finding that SC decreases stress shielding and bone resorption in the proximal periprosthetic bone, our results show that SC does not compromise initial implant stability, thus providing a strong case for SC over FC.

**Conclusions** Although a long term follow-up study is required to confirm the reliability of the technique and to observe natural history of radioluncency lines, our results are encouraging, since many studies reported that most of aseptic failures in TKA occur in the early postoperative period. The presence of radiolucency lines was significantly associated with age > 75 years. Therefore, we do not recommend using surface cementation and mobile trays in these patients.

### ORAL COMMUNICATIONS

### *C38–ACUTE CAPSULO-LIGAMENTOUS INJURIES 1*

### Results of soft tissue constraint reconstruction in complex elbow instability

G. Giannicola*, D. Polimanti, F. Sacchetti, M. Scacchi, A. Greco, F. Postacchini

“Sapienza” Università di Roma (Rome, IT)

**Introduction** Soft tissue constraint lesions (STCL) are frequent in complex elbow instability (CEI) and the repair is fundamental to restore elbow stability. Aims of this study are to describe a standard surgical technique to repair soft tissue constraint lesions in CEI based on a clear identification of the pathoanatomy and to report clinical prospective results of this surgical technique.
**Materials and methods** There were 45 elbows with CEI. The mean age was 54 (range 22–75) years. Surgery consisted of anatomical reduction and stable internal fixation (ORIF) of all fractures and replacement of Mason III radial head fractures. The repair of soft tissue injuries was carried out using: (1) double wire suture anchors (Twinfix 5 mm, Smith & Nephew, Memphis, TN, USA) with modified Mason-Allen stitch for ligament, capsule and tendon detachments; (2) side to side cross sutures for ligament/tendon midsubstance tears. The mean follow-up was 25 months. MEPS, m-ASES, DASH score were used for clinical evaluation. At 6 months follow-up, elbow stability was evaluated with varus and valgus stress tests, pivot-shift test and drawer test; in patients with positive or uncertain tests a fluoroscopic evaluation was performed.
**Results** The average extension loss was 10 (0–50) and the average flexion was 139 (90–145); forearm rotation averaged was 82 (5–90) of pronation and 80 (0–90) of supination. The functional arc of motion (30–130 in E/F and 50–50 in P/S) was achieved in 39 out of 45 patients. The average MEPS score was 94 (70 to 100); according to MEPI, there were 34 excellent, 9 good, 2 fair results. The average DASH score was 5.6 (0–38.8); the average ASES score was 89 (64–100). 42 patients had no sign or symptom of elbow instability at the last follow-up while 1 patient had a moderate posterolateral instability and 2 a mild varus instability.
**Discussion** The recognition and treatment of capsule-ligamentous and muscle-tendinous injuries represent two essential steps in the diagnostic and therapeutic algorithm of complex elbow instability (CEI). After ORIF, a standard technique consisted in ligament reinsertion with suture anchors and modified Mason-Allen stitch associated with cross sutures of midsubstances tears, leads to satisfactory clinical results and recovery of elbow stability in most of cases.
**Conclusions** This is the first study reporting a validated surgical technique to repair STCL based on a clear identification of its pathoanatomy.

### Results in arthroscopic treatments after acute dislocation compared with results in chronic dislocation repair

V. Piccinni*[1], V. Campagna[1], P.I. Falco[1], M. Marchitiello[1], G. Rotundo[1], S. Gumina[2]

[1]Policlinico Militare (Rome, IT);
[2]Università La Sapienza (Rome, IT)

**Introduction** Recent studies have demonstrated that the arthroscopic treatment after first dislocation of the shoulder can improve the percentage of good results, decrease the number of further dislocation, avoid the open treatment and the evolution in chronic instability. The treatment of the first episode is more indicated in young people, with high request for sport and work activity. The aim of this paper is to compare the result of the arthroscopic treatment after first dislocation with those of multiple dislocations.
**Materials and methods** From 2004 to 2009 we treated arthroscopically two groups of patients. The first group, after single dislocation, 63 patients with a mean age of 21 (range 18–24), with high sports performances and work demand; in the second group 90 patients with a mean age of 28 (range 17–40), after multiple dislocations, with no demand of high performance of the shoulder. All the patients were studied with X-rays and MRI pre-operation exams. All patients were evaluated with Rowe Scale Evaluation schedule. Minimum follow up was 2 years and the post-operative protocol was the same for the two groups.
**Results** Follow-up ranged from 2 to 8 years. In the first group good results were the 90.8 %, in the second 84.5 %. No rate of re-dislocation in the first group, 2 patients (9.2 %) had moderate instability and apprehension and they did not return to the previous level of sport and work performance. In the second group we reported bad results in the 15.5 % with the rate of re-dislocation of 8 %. We reported higher percentage of slap lesion in the first group (23.8 %) compared with the second one (11.1 %).
**Discussion** Two important factors were reported as correlated with the increase of anatomical damages in the first group: the high energy trauma and the surgical timing after the single episode. In the second group we reported worse capsular tissue quality, higher rate of “engaging Hill Sachs lesions” (16 %), not observed in the first group. In the first group we observed more damages in the anterior and superior capsular structures, in the second group more anterior and inferior damages.
**Conclusions** The arthroscopic treatment after first dislocation is associated with high percentage of good results, it can decrease the rate of re-dislocation in young active patients with high request of sport and work performance. This is a sure indication in this population. We can avoid the evolution in chronic instability and decrease damages of the anatomical structures.

### P.C.L. avulsion: comparison between two techniques

M. Giaracuni*, M. Valeo, D. Attala, G. Bellacozza, M. Gurzì, L. Di Martino, C. Villani

Dipartimento di Scienze dell’Apparato Locomotore, Policlinico Umberto I, Università di Roma La Sapienza (Rome, IT)

**Introduction** The P.C.L. avulsion is an uncommon fracture and his treatment is discussed in the literature. We compared two techniques: open reduction (with direct internal synthesis device or non-absorbable wire-suture) and arthroscopical reduction with mini open incision. In the last years, according to several authors, the arthroscopic technique seems to give the best result and it is considered the gold standard.
**Materials and methods** We compared two groups of patients, each group was composed of one man and one woman. The range of age was 35–45. Every patient was diagnosed with isolated P.C.L avulsion and as treated for days after trauma. One group was treated with open via access, end to end suture and cancellous screw, the other group was treated with AP and AL access through a transeptal arthroscopic via and a metal button was used to fixate the tendon.
**Results** The two patients treated with open via complained post-operative pain. Therefore for this first group a period of 15 days without weight bearing and with locked knee brace at full extension was planned. Conversely, the group treated with transeptal arthroscopic via began immediately weight bearing with knee brace and passive motion from 0° to 90°. The two groups were evaluated after 3 months. The group treated with open via had a 30° of motion deficit compared to contralateral knee, the group treated with transeptal arthroscopic via had 10° of motion deficit. All patients achieved an anatomical reduction and complete *restitutio ad integrum* of the avulsion-fracture with full return to daily activity.
**Conclusions** At follow-up, considering the objective and subjective data, the arthroscopic transeptal technique with AP fixation and endobutton resulted the less traumatic treatment and the best borne by the patient. Therefore, despite the small number of the samples, this study shows that both groups of patients had almost complete healing and arthroscopic transeptal via demonstrated clear advantages in the treatment of P.C.L. avulsion.

### The repair of ACL in acute and chronic phases

G.L. Canata*, V. Casale, A. Chiey, D. D'Elicio

Centro Traumatologia Sport Koelliker (Turin, IT)

**Introduction** Literature does not agree on the possibility of repairing an ACL lesion in an acute or chronic phase. In proximal lesions, a ligament may sometimes be reinserted. We compared a group of subjects treated in the acute phase (within the first 3 weeks) and a group of subjects treated in the chronic phase (after 4 weeks).
**Materials and methods** 37 patients were evaluated, with repair of the ACL for a proximal lesion (equal to 4 % of the subjects operated on the ACL at the same time). They were divided into 2 groups. Group A: operated within 3 weeks of the trauma, 25 patients, 10 males and 15 females; mean age 26 years (range, 12–41); meniscal lesions in 8 cases and chondral ones in one case. Group B: 17 patients, 11 males, 6 females; mean age 33 years (range, 12–70); meniscal lesions in 4 cases and chondral ones in 3. In all of the patients, a transosseus reinsertion of the ACL with cortical fixation was performed. A pre- and post-operative evaluation using the KOOS, IKDC, Lysholm and Tegner scores was carried out. Statistical analysis with *t* test. Mean follow-up was 42 months.
**Results** The mean preoperative KOOS score: Group A 50 (St Dev 17), Group B 59 (St Dev 19). Postoperative Group A 98 (St Dev 7), Group B 97 (St Dev 16). Mean IKDC score: postoperative Group A 95, Group B 95. Lysholm postoperative Group A 99, Group B 96. Tegner Group A: preoperative score 7, postoperative 7; Group B preoperative 7 and postoperative 7. Significant differences do not emerge between the two groups.
**Discussion** ACL repair is only possible in the proximal lesions with good quality ligaments. The time since the trauma does not determine the deterioration of the results. A reparative act is possible in a modest percentage of cases, but it allows the recovery of previous activities in the majority of cases.
**Conclusions** ACL repair is possible in a limited number of cases. The proximal lesion site and the quality of the ligamentous tissue have more importance than the time since trauma.

### Healing response technique versus augmentation using semitendinosus in partial tear of ACL: clinical and biomechanical differences

G. Cerulli*[1], S. Palladini[2], E. Sebastiani[3], G. Placella[3], G. Potalivo[1]

[1]Nicola's Foundation Onlus, Let People Move Research Institute (Arezzo, IT);
[2]Università degli Studi (Perugina, IT);
[3]Clinica Ortopedica e Traumatologica, Università degli Studi (Perugia, IT)

**Introduction** Partial tears of the anterior cruciate ligament (ACL) continue to be an issue in scientific literature as there is still no gold standard regarding surgical treatment. The aim of our study is to evaluate the healing response technique in partial tears of the ACL in young and active patients and compare the results with those obtained using the reliable and validated technique with hamstring augmentation.
**Materials and methods** 30 patients, average 22 yrs, with I and II degree lesions according to the Hugston ACL score were randomized into two groups: group A (15 pts.) underwent the healing response technique; group B (15 pts.) underwent the hamstring augmentation technique. All surgical procedures were performed by the same person. Both pre-and post operative subjective, clinical and biomechanical measurements were performed using: the S.A.N.E. and Lysholm scores and the anterior tibial translation using the KT-2000 knee arthrometer. Average follow-up was 8 months.
**Results** All the outcomes measured showed a great improvement post-operatively. Group A went from an average S.A.N.E score of 44.67 and average Lysholm score of 38.33 to an average score of 93.33 and 92.33, respectively. The average pre-op KT-2000 measurements were 2.96 mm in group A and 3.1 mm in group B. At follow-up the average values recorded were 0.7 mm and 0.6 mm, respectively.
**Discussion** Surgical treatment of ACL partial ruptures can involve either reconstruction or repair. Augmentation with the semitendinosus enables immediate recovery of joint stability but it presents the disadvantages of a biological reconstruction. The healing response is a biological stimulation technique which by means of microfractures supplies growth factors to the original ligament thus recovering knee function.
**Conclusions** No statistically significant differences between the two groups were observed. Therefore we suggest that the Healing Response Technique could be a valid alternative treatment for this type of lesion in this type of patient.

### *C39–ARTHROSCOPY*

### Autologous chondrocyte implantation and microfractures associated to high tibial osteotomy in the treatment of varus knee with severe osteoarthritis: a clinical study at 10-year follow-up

A. Ferruzzi*, R. Buda, A. Timoncini, S. Giannini

II Clinica, Istituto Ortopedico Rizzoli (Bologna, IT)

**Introduction** High tibial osteotomy represents a viable alternative to prosthetic knee replacement in the treatment of middle-aged active patients with medial osteoarthritis and varus knee. Despite good results reported at mid-term follow-up, long term series show a deterioration of the clinical and radiographic outcome over time. Several authors have therefore suggested an association with cartilage repair procedures in order to improve the long-term results. Aim of the study was to verify the effectiveness and utility of autologous chondrocyte implantation or microfractures associated to high tibial osteotomy, in the treatment of varus knee with severe osteoarthritis.

**Materials and methods** Between 1999 and 2002, 70 patients affected by varus knee deformity with symptomatic medial osteoarthritis, rated III–IV according to the Kellgren-Lawrence scale, a ROM with at least 90° of flexion, age less than 60 years, were selected for the study. Patients were randomized in three groups: 24 were treated by isolated high tibial osteotomy, in 22 cases arthroscopic autologous chondrocyte implantation was associated to the osteotomy, while the remaining 24 were treated with high tibial osteotomy and microfractures. A clinical assessment following the HSS and WOMAC rating score and a radiographic study with standard radiographs and MRI scan was performed preoperatively, serially postoperatively and at the final follow-up. A statistical analysis with the Wilcoxon test, the Mann–Whitney test, the paired T test, the One Way ANOVA test, the Pearson's and the Spearman's correlation was carried out to test the significant differences between baseline and follow-up measurements.
**Results** Clinical and radiographic evaluation performed preoperatively, at 6 month, 3 and 10 years post-operatively, showed no statistically significant differences in the three groups. At 1 year follow-up the patients treated with tibial osteotomy associated to microfractures, compared to the other patients, showed the worst clinical and radiographic results ($p < 0.005$).
**Discussion** In order to enhance the tissue regeneration and improve the long term outcome, cartilage repair procedures have been suggested to be associated to high tibial osteotomy in the treatment of varus knee with severe gonarthritis. In our study associated cartilage reparative or regenerative procedures did not provide an improvement in clinical and radiographic results.
**Conclusions** Autologous chondrocyte implantation and microfractures procedures did not provide an improvement in clinical and radiographic results after high tibial osteotomy for the treatment of varus knee with severe gonarthritis.

### Meniscal suture with the "Mulberry" technique: outcomes at 3-year follow-up

G. Cerulli*[1], F. Fantasia[2], G. Placella[3], E. Sebastiani[3], G. Potalivo[1]

[1]Nicola's Foundation Onlus, Let People Move International Orthopedic and Traumatology Institute (Arezzo, IT);
[2]Dipartimento di Ortopedia, Nuova Clinica San Francesco (Foggia, IT);
[3]Clinica Ortopedica e Traumatologica, Università degli Studi di Perugia (Perugia, IT)

**Introduction** The meniscus is a structure of fundamental importance for the long term health of the knee. Meniscal injuries are very common and they increase the risk of developing gonarthrosis over the years.
**Materials and methods** Fifteen patients were enrolled in our study; average age at surgery 22.1 years. All had a diagnosis of meniscal lesion associated with anterolateral instability due to ACL tear. All procedures were performed by the same surgeon. The medial meniscal injuries were treated using the out-in "Mulberry"-type technique. The surgical reconstruction of the ligament was done using the All-Inside technique. At 3-year follow-up patients were revaluated clinically by means of the Lysholm Knee Scale, the Tegner Activity Level Scale, the VAS and the KOOS Knee Survey as well as undergoing biomechanical functional tests.
**Results** No recurrent meniscal lesions were recorded; all patients presented a complete ROM and the McMurray and Appley tests were negative. The average VAS score was 1.2 compared to the preoperative 5.1. The average Lysholm score was 90.4. Average results with the KOOS system: 97.6 for pain, 93.3 for general symptoms, 99.9 for everyday life activities, 93.6 for sport and 79.8 for the quality of life. The Isokinetic evaluation with Kin-Com revealed an increase in the peak force of the operated knee's extensors and flexors compared to the contralateral knee.
**Discussion** Total lateral meniscectomy causes a 40–50 % reduction in surface contact with an increase in stress of the lateral compartment up to 200–300 % compared to normal. Partial meniscectomy significantly alters the ability of the meniscus to bear the load; the loss of the internal third of the meniscus increases by about 65 % the contact pressure between the femoral condyle and the tibial plateau. It is therefore clear why it is important to preserve the meniscus to avoid joint damage in time, in particular the onset of osteoarthrosis of the knee.
**Conclusions** The "Mulberry"-type out-in technique guarantees very satisfactory results both from a clinical and a functional point of view, with a relatively low failure rate considered the number of patients treated; furthermore this technique combines efficacy, simplicity, rapidity as well as a lower risk of iatrogenic chondral lesions, neurovascular complications and infections.

### *C40–TRAUMATOLOGY 5*

### Dilops: a new elastic osteosynthesis in pertrochanteric femoral fractures fixation

P. Palombi*[1], A. Palombi[2]

[1]Ospedale C.T.O. (Rome, IT);
[2]Università Tor Vergata (Rome, IT)

**Introduction** It is introduced the divergent locked screws system for the treatment of pertrochanteric fractures (AO/OTA 3.1.A1 and A2): it is characterized by the biomechanical advantage of the shortening of the lever arm that stands between the gravitary axis bearing on the femoral head and the fulcrum of the osteosynthesis system, allowing a robustness osteosynthesis reached by mini-invasive approach. Surgical technique is shown. Aim of this paper is to evaluate the new system and compare its preliminary results with the current literature. At the time of this paper is possible to comment preliminary and midterm results.
**Materials and methods** Between July 2009 and January 2012, 229 patients were admitted for proximal femoral fracture (AO/OTA 31.A1 and 31.A2) in our Center. One hundred seventy of them (109 women and 61 men) were recruited in a prospectic study and treated with divergent locked screws system. Clinical and radiographic follow-up was made at 0, 2, 6, 12, 24 weeks, and then at 12 and 18 months. Results were evaluated by objective and subjective scores. Objective scores were assessed by orthopaedic surgeons on the basis of clinical and radiographic assessment, intra- and post-operative blood loss, operative time, intraoperative X-rays exposition, rate of complications and failure, time to the full weight bearing. Subjective scores were assessed by surgeons and patients on the basis of restored function, restored quality of live and overall satisfaction.
**Results** Nearly 85 % of patients who were alive after 12 months showed good or excellent results. Worst results were associated with age > 85 years old, high comorbidity and cognitive diseases.
**Discussion** The divergent locked screws system showed to be a powerful and cost-effective alternative in the treatment of stable (AO/OTA 31.A1) and unstable (AO/OTA 31.A2) pertrochanteric femoral fractures. It showed easy intraoperative management of fracture fragments, very good stability, and rapid bone healing.
**Conclusions** The divergent locked screws system showed to be a powerful and cost-effective alternative in the treatment of stable (AO/OTA 31.A1) and unstable (AO/OTA 31.A2) pertrochanteric femoral fractures. It showed easy intraoperative management of fracture fragments, very good stability, and rapid bone healing.

### X-ray screening in Whiplash injury of cervical spine in a Trauma Care Unit after car accident: diagnostic supplement or bad habit?

A. Bistolfi*[1], M. Cerrato[2], C. Gaido[2], I. Carnino[2], A.M. Federico[2], M. Paonessa[1], F. Rosso[2], G. Massazza[3]

[1]AO CTO M. Adelaide (Turin, IT);
[2]Università degli Studi (Turin, IT);
[3]AO CTO M Adelaide, Università degli Studi (Turin, IT)

**Introduction** In Emergency Units, cervical spine X-rays are frequently used to exclude bone injuries in head and neck traumas. In more than 95 % of patients without neurological deficits, the exam results negative for clinically serious lesions. That is the reason for the great increase of waiting time in trauma care units, of sanitary costs, of the over-crowding in radiology departments. The exposition to X-rays is not justified by a diagnostic usefulness in most of the cases.
**Materials and methods** We searched in literature the indications to perform cervical spine X-rays after Whiplash injury (head and neck trauma) with the following key-words: cervical spine and radiography; cervical spine trauma; cervical spine injury AND nexus; cervical spine AND canadian C-spine rule in the last 10 years.
**Results** There are not validated guidelines about this topic, neither in Italy, nor abroad. The application of clinical scales has been proposed to reduce the number of radiographies after soft traumas. These scales can identify with very high probability patients to be safe from injuries and the cases that do not need the X-ray control. We found out two scales: the NEXUS and the Canadian C-Spine rule (CCR). These scales have high sensibility to identify the cases in which X-ray control is uselessness. In major traumas, such as the high energy ones or when neurological deficit symptoms coexist, X-rays are recommended to find out clinically important injuries: fracture, dislocation, ligament instability.
**Discussion** The possibility to focus on the necessary radiographic exams following guidelines in trauma care units would permit to uniform the medical approach to head traumas. In this way the approach would become faster and more effective. There would be economical advantages both for the sanitary structure and for the patients. Furthermore we could reduce waiting times, the over-crowding of radiology departments in trauma care units, the ipermedicalization and the exposition to non essential X-rays. In Italy X-ray control, even if don't diagnostic, is used to support indemnification requests. This habit has been already abolished in most of the abroad countries.
**Conclusions** Standardization in the use of the X-ray control would bring advantages to the patients and the sanitary structures. The working out of specific guide lines is recommended. The consensus of the sanitary structures is essential to avoid legal medicine problems.

### Comparison of different surgical methods for calcaneus fractures: nosological classification and clinical results evaluation

L. D'andrea, F. Furci, E. Foci, S. Mauro, V. Ioli, M.A. Rosa*

Scuola di Specializzazione in Ortopedia e Traumatologia, Sezione di Ortopedia e Traumatologia, Dipartimento Specialità Chirurgiche, Università degli Studi di Messina (Messina, IT)

**Introduction** Fractures of the calcaneus occur due to falls from a height and/or road accident trauma (85 %). They represent 2 % of all fractures and have a highly significant economic importance. Several methods of treatment are described in literature.
**Materials and methods** The authors present their experience conducted on 40 patients, 28 M and 12 F with an average age of 54 years. They were treated at the UOC of Orthopaedics and Traumatology Hospital "G. Martino", Messina from 1 November 2003 to 31 March 2010, with a follow-up of from 2 to 7 years. The authors have used the CT classification of Sanders (1992), based on location and the number of lines of fracture of the posterior articular surface, and that of Utheza (2001), which describes the thalamic fractures of the calcaneus with 3D CT. Several fixation techniques were used: ORIF with plates and screws (7 patients), closed reduction and percutaneous synthesis with Kirschner wires (13 patients) or cannulated screws (20 patients). In 11 patients bone grafting was also needed. We used the Maryland Foot Score for clinical evaluation. X-rays, CT and baropodometry were used for the instrument evaluation.
**Results** We had the best results when good reduction of the subtalar back, good restoration of joint motility and correct longitudinal alignment of the heel and front were obtained. According to AA the surgical technique of reduction and percutaneous fixation with cannulated screws ensured a more rapid functional recovery of the patient than other methods. No complications of surgical wounds or deep and/or superficial infectious processes were observed. The association with ankle fracture with clamp diastasis resulted in a delayed decision for weight bearing.
**Discussion** The choice of surgical treatment is determined by the age of patient, functional demands, type of fracture and eventual correlated joint involvement, associated comorbidities and surgeon's experience. The analysis of literature provides evidence that the various methods of synthesis guarantee good results with a low rate of complications.
**Conclusions** In our experience, the accuracy in the restoration of normal relations of the hindfoot joints during surgery is an important element in determining the degree of post-operative functional recovery. The use of cannulated screws, where possible, minimizes the rate of possible local complications, reduces the time of surgery and the risk of superinfection, and it is associated with the best functional score in follow-up.

### *C41–HIP 5*

### Hospital cost of dislocation after hemiarthroplasty, total hip arthroplasty and revision prosthesis

M. Marinelli[1], R. Procaccini*[1], I. Ponzio[1], A. Soccetti[1], L. De Palma[2]

[1]Ospedali Riuniti (Ancona, IT);
[2]Cattedra Ortopedia e Traumatologia (Ancona, IT)

**Introduction** The treatment of dislocation following primary hemiarthroplasty (HA), total hip arthroplasty (THA) and revision arthroplasty (RP) involves the use of expensive hospital resources and sometimes also revision surgery. The hospital costs associated with treating this complication have not been previously analysed, to our knowledge, in the context of a European public hospital. The purpose of this study was to assess the financial impact of treating dislocations at our institution.
**Materials and methods** Between October 2001 and August 2009, 2014 consecutive hip replacements were performed by 18 surgeons at our institution. The data of all patients were retrieved from the operating room database and mined for implants treated for dislocation within 6 weeks. There were 87 prostheses (18 HA, 44 TH and 25 RP). The cost of treating these dislocations was assessed by determining the cost of each procedure required to re-establish hip stability and expressed as percent cost increase compared with uncomplicated HA, THA and RP.
**Results** Of the 87 hips that sustained early dislocation, 35 remained stable after one or more closed reductions and 52 underwent one or more closed reductions but ultimately required revision surgery. Each dislocation treated with closed reduction, open reduction and closed reduction and revision surgery considerably increased the cost of the HA, THA and RP.
**Discussion** Dislocation after HA, THA and RP continues to be a prevalent and costly complication that diminishes the cost-effectiveness of an otherwise very successful surgical procedure.

### Periacetabular osteotomy with neurophysiological monitoring

V. Bellotti*, J. Climent, E. Astarita, F. De Meo, C. Cardenas, M. Ribas

Instituto Universitario Dexeus (Barcellona, SP)

**Introduction** We describe the use and utility of neurophysiological monitoring during mini invasive trans-sartorius periacetabular osteotomy for prevention of lower limb's nerve lesions.
**Materials and methods** We describe the use of neurophysiological monitoring during a mini invasive periacetabular osteotomy in a series of 40 consecutive cases.
**Results** It was possible monitoring potentially iatrogenic damages depending on the different osteotomy steps.
**Discussion** The neurophysiological monitoring during a periacetabular osteotomy is an effective technique to prevent iatrogenic damages during the different osteotomy steps and acetabular redirectioning.
**Conclusions** The neurophysiological monitoring during a mini invasive trans-sartorius periacetabular osteotomy is a valid aid on nerve injury prevention, it allows monitoring in a selective way the potentially iatrogenic damages on the different osteotomy steps.

### The space femoroacetabular impingement: definition and treatment

V. Bellotti*, E. Astarita, F. De Meo, C. Cardenas, M. Ribas

Instituto Universitario Dexeus (Barcellona, SP)

**Introduction** The femoroacetabular impingement space (FAI) is the most frequent type of impingement of the hip. We describe the etiology, the diagnosis, and the treatment based on the experience of many years at a national center of reference in the treatment of diseases of the hip.
**Materials and methods** A retrospective study of over 400 cases operated by arthroscopy, mini open technique, and by safe open dislocation over a period of 8 years.
**Results** We describe the results of treatment in the medium term, and the rate of success depending on the preoperative degenerative stage of the hip joint.
**Discussion** The FAI space is a mechanical condition and its treatment is surgical. The current proposed surgical techniques are able to correct and treat this condition. The preservation or suture of the labral structure or, in defect, its reconstruction with a graft, seems to promote a higher and stable clinical outcome over the time. The open technique seems to offer a major correction accurancy of both femoral and acetabular defects.
**Conclusions** In this series of over 400 cases of FAI space operated in a period of time of 8 years we observed that satisfactory results can be obtained by the actual proposed techniques. Results on the medium-term seem to depend more from preoperative degenerative stage than from the type of surgical technique.

### *C42–BASIC SCIENCE 1*

### Biomolecular investigations in osteoarthritis

M. Saporito*[1], V. Bravata[2], L. Minafra[2], F. Cammarata[2], S. Caldarella[2], F. Boniforti[3]

[1]Università degli Studi di Palermo (Palermo, IT);
[2]Lab. Tecn. Oncol. LATO (Cefalù, IT);
[3]Fondazione San Raffaele Giglio (Cefalù, IT)

**Introduction** The classification of OA is based mainly on clinical and radiographic evaluations. Proteogenomic investigations have taken a significant role in the aetiology of OA. Aim of the study was to correlate clinical and radiographic grading of OA with proteogenomic data.
**Materials and methods** We enrolled 87 patient candidates for knee surgery with arthroscopy or arthroplasty. Patients were divided into two age groups: 54–0 and 71–86. Clinical evaluation was performed using Knee Society's score. The radiographic evaluation was performed with the KL scale on antero-posterior and lateral views of the knee. Genotyping analysis was performed using blood samples and was expressed into 3 types: wild-type genotype in both alleles, polymorphism in one allele and polymorphism in both alleles. Proteogenomic analyses were performed using urine, synovial fluid and synovium samples. Clinical, radiological and genotyping data obtained were processed using statistical correlation's algorithms.
**Results** The A group consisted of 24 patients, 17 Y and 7 O. The KS was poor in 13 cases and fair in 11 cases. The B group consisted of 21 patients, 14 Y and 7 O. The KS was poor in 19 cases and fair in 3 cases. The C group consisted of 22 patients, 11 Y and 11 O. The KS was poor in all 22 cases. Each test was associated with a genotype panel of each analyzed gene.
**Conclusions** This study will facilitate the understanding of the molecular mechanisms involved in OA and allows a better definition of the grading of the pathology.

### Biomechanics of the goat three bundle anterior cruciate ligament

M. Ronga*[1], T. Tischer[2], P. Smolinski[3], F. Fu[3]

[1]Dipartimento di Ortopedia e Traumatologa, Università dell'Insubria (Varese, IT);
[2]Department of Orthopaedic Surgery, University of Rostock (Rostock, DE);
[3]Department of Orthopaedic Surgery, University of Pittsburgh (Pittsburgh, USA)

**Introduction** The goat is a widely used animal model for basic research on the anterior cruciate ligament (ACL), but the biomechanical role of the different bundles [intermediate (IM), anteromedial (AM), postero-lateral (PL)] of the ACL is unclear. Therefore, the aim of this study is to describe the biomechanical function of the different bundles and evaluate its use for a double bundle ACL reconstruction model.
**Materials and methods** A CASPAR Stäubli RX90 robot with a six degree-of-freedom load cell was used for measurement of anterior tibial translation (ATT) (mm) and in situ forces (N) at 30° (full extension), 60°, 90° as well as rotational testing at 30° in 14 paired goat knees before and after each bundle was cut.

**Results** When the AM-bundle was cut, the ATT increased significantly at 60° and 90° of flexion ($p < 0.05$). When the PL-bundle was cut, the ATT increased only at 30°. However, most load was transferred through the big AM-bundle while the PL-bundle shared significant load only at 30°, with only minimal contribution from the IM-bundle at all flexion degrees.

**Discussion** The observed biomechanical results in this study are similar to the human ACL observed previously in the literature. Though anatomically discernible, the IM-bundle plays only an inferior role in ATT and might be neglected as a separate bundle during reconstruction.

**Conclusions** The goat ACL shows some differences to the human ACL, whereas the main functions of the ACL bundles are similar.

### The influence of medial patello-femoral ligament on patello-femoral joint kinematics

T. Bonanzinga*[1], B. Sharma[1], S. Bignozzi[1], F. Colle[1], G.M. Marcheggiani Muccioli[1], F. Raggi[1], D. Dejour[2], S. Zaffagnini[1]

[1]Istituto Ortopedico Rizzoli (Bologna, IT);
[2]Lyon Ortho-Clinic (Lyon, FR)

**Introduction** The aim of this study was to study the morphology of the Medial Patello-Femoral Ligament (MPFL) and its influence on patello-femoral kinematics with reference to the trochlea Hypothesis: The MPFL is only a restraint during motion, against a load inducing lateral shift, functioning as an aponeurosis, to guide the patella through a trochlear groove.

**Materials and methods** We performed a kinematic study of six cadaveric knees, three of each side, through passive 10°–120° of flexion and extension, with centre point of patella as a reference point over posterior condylar and mechanical axis of the femur, by a non-image based navigation system, under an axial quadriceps load of 60 N, with free tibial rotation and eliminated femoral anteversion; with or without a laterally directed load of 25 N, at 0°, 30°, 60° and 90°; and with native MPFL and with MPFL cut.

**Results** MPFL femoral insertion noted to be variable. In comparison to the MPFL-intact state, the patella shifted laterally in MPFL-deficient state, even without laterally directed load. The variability in kinematics could not be explained on the basis of variation in trochlear morphology. MPFL was anisometric, the insertion points of the inferior bundles coming closer in flexion.

**Discussion** Reported results are comparable to thosre published by Baldwin et al. [1] concerning the patella lateral shift in MPFL-deficient state, but they are different from what reported by Sandmeier et al. [2] in different experimental conditions.

**Conclusions** While, MPFL may guide the patella shift and tilt during knee motion, in normal knee trochlear morphology does not influence kinematics. The ligament act as a passive restraint and its complex anatomical structure allows it to be anisometric during full range of motion.

**References**

1. AJSM (2009) 37:2355–23619
2. AJSM (2000) 28(3):345–349

### Minced umbilical cord fragments as a potential cell source for orthopaedic tissue engineering: an in vitro study

A. Marmotti[1], S. Mattia[2], F. Dettoni[1], M. Bruzzone[1], D.E. Bonasia[1], D. Blonna[1], F. Castoldi, R. Rossi[1], C. Tarella[2], G.M. Peretti[3]

[1]Dipartimento di Ortopedia e Traumatologia, Università degli Studi di Torino (Turin, IT);
[2]M.B.C.-Molecular Biotechnology Center, Università degli Studi di Torino (Turin, IT);
[3]Department of Sport, Nutrition and Health Sciences, University of Milan (Milan, IT)

**Introduction** A promising approach for musculoskeletal repair and regeneration is the use of mesenchymal stem cell (MSC)-based tissue engineering. Bone marrow is the most commonly employed source of MSCs. The Umbilical Cord (UC) seems a promising alternative of allogeneic MSCs due to its virtual "unlimited "availability. The aim of the current study was: (1) applying a simple protocol based on mincing the umbilical cord (UC), without removing any blood vessels or using any enzymatic digestion, to rapidly obtain a consistent number of multipotent UC-MSCs; (2) verifying the phenotype of these cells; (3) investigating the possible differentiation toward osteoblastic-chondroblastic-mioblastic-adipoblastic lineage.

**Materials and methods** Donors: 6 Fresh UC samples were retrieved at the end of caesarean deliveries. Samples were manually minced into fragments < 4 mm length and cultivated in an MSC-expansion-medium. At day 14, UC tissue was removed and adherent cells were allowed to expand for 2 additional weeks. At day 28, adherent cells were collected and replated until confluence was reached (Passage 1 or P1). Cell counts, Immunophenotypic characterization, Fluorescence-In Situ-Hybridization (FISH), telomere analysis, T-lymphocyte-Immunosuppression and multilineage differentiation (EUROMED-Osteogenic-Differentiation-Kit, EUROMED-Chondrogenic-Differentiation-Kit, DMEM-ß–mercaptoethanol-FGF, EUROMED-Adipogenic-Differentiation-Kit) were performed in UC-MSCs at P1 or P2.

**Results** At P1, we obtained a mean value of $22.8 \times 10^6$ cells (SD 1.7) from each UC, corresponding to 0–68 $\times 10^6$cells/gram of UC. At immunophenotypic characterization, cells were positive for CD73, CD90, CD105, CD44, CD29, HLA-I, and negative for CD34 and HLA-class II, with a subpopulation that was negative for both HLA-I and HLA-II. Results from FISH demonstrated that 95–100 % of UC-MSCs were of fetal origin. Telomere length of UC-MSCs was similar to that of Bone Marrow (BM) MSC from young donors (aged 20–30 years). At 5 days, the supernatant of UC-MSC cultures had immunosuppressive activity upon T-Lymphocyte cultures. The mixed UC-MSC population was able to differentiate towards osteogenic, adipogenic, miogenic and chondrogenic lineages after culture using commercial differentiation media. In particular, differentiation towards chondrogenic lineage was observed both in pellet cultures and in tri-dimensional scaffold cultures (Chondrogide and HYAFF-11).

**Discussion** These results suggest that the straightforward procedure of collecting UC-MCS at P1 from minced umbilical cord fragments can achieve a valuable cell population. The obtained UC-MSCs seem to have the potential to be good candidates for tissue engineering applications in orthopaedics.

**Conclusions** The concept of this study may indeed be considered as a future hypothetical option for patients who might benefit from stem cells therapy.

### Cell outgrowth from chondral fragments into a composite scaffold: a potential mechanism for "one stage" cartilage repair? An in vitro study

A. Marmotti[1], D.E. Bonasia[1], M. Bruzzone[1], D. Blonna[1], F. Dettoni[1], F. Castoldi[1], R. Rossi[1], C. Tarella[2], G.M. Peretti[3]

[1]Dipartimento di Ortopedia e Traumatologia, Università degli Studi di Torino (Turin, IT);
[2]Dipartimento di Ematologia, Università degli Studi di Torino (Turin, IT);
[3]Department of Sport, Nutrition and Health Sciences, University of Milan (Milan, IT)

**Introduction** Minced cartilage fragments provide a viable cell source for one stage cartilage repair. Human in vitro explant cultures show some limitations (i.e. reduced cell migration and outgrowth from cartilage fragments, compared to animal models) when compared to that of animals. Aim of the study is: (1) verifying chondrocyte migration/outgrowth from human cartilage fragments and if TGF-beta and G-CSF exposure could enhance cell migration; (2) investigating some of the possible cellular mechanisms behind this phenomenon.
**Materials and methods** Cartilage fragments construct preparation: articular cartilage from 25 human knees (< 35 years) was harvested and minced into small fragments; a non-woven esterified HA derivative felt (HYAFF-11) was cut to pieces of 0.7 $cm^2$; two sheets were put in a culture dish one atop the other and homogeneously hydrated with 100 ul of human PRP; 10–15 mg of cartilage fragments were evenly seeded atop the membrane and retained with a coating of approximately 200 ul of commercial fibrin glue; constructs were cultured for 1 month both in standard culture medium and under exposure to G-CSF (10 ng/ml) and/or to TGF-beta (10 ng/ml). Explant cultures were evaluated histologically and with immunofluorescence.
**Results** Compared to unstimulated cultures ($p < 0.05$), chondrocyte outgrowth at 1 month increased in a mean ratio of 1,7:1 with exposure to TGF-beta, in a mean ratio of 1.9:1 with exposure to G-CSF and in a mean ratio of 1,8:1 with exposure to both factors. No statistical differences were observed between exposure to G-CSF or TGF-beta or both factors ($p > 0.05$). Immunofluorescence of migrating cells was positive for sox9, CD151, CD49c and negative for CD105, consistent with a predominant chondrogenic phenotype; G-CSF Receptor was detected on migrating cells with immunofluorescence; exposure to G-CSF slightly decreased SOX-9 expression and increased PCNA and beta-catenin expression in migrating cells.
**Discussion** G-CSF and TGF-beta exposure improves chondrocytes outgrowth from human cartilage fragments loaded into a HA/fibrin/PRP scaffold. Efficiency of migration is not increased if TGF-beta is added to G-CSF during construct culture. The changes in expression of Sox-9 (slightly decreased) and PCNA and beta-catenin (increased) under G-CSF exposure suggest a proliferative phenotype of cells, similar to the chondrocytes from the Superficial Zone and to the prechondroblastic cells of early stage of chondrogenesis.
**Conclusions** This supports a possible role of G-CSF in increasing chondrocyte outgrowth during in vivo one stage cartilage repair with minced human cartilage fragments.

### *C43–ACUTE CAPSULO-LIGAMENTOUS INJURIES 2*

### Surgical versus conservative treatment after acute patellar dislocation: a systematic review and meta-analysis

M. Saccomanno*, L. Deriu, S. Careri, C. De Ieso, F. Donati, G. Milano, C. Fabbriciani

Università Cattolica del Sacro Cuore, Istituto di Clinica Ortopedica (Rome, IT)

**Introduction** Acute patellar dislocation accounts for 2–3 % of all knee injuries; nevertheless if it's not correctly treated, it can result in patellar instability, decreasing of activity level and patella-femoral osteoarthritis. The treatment of the first dislocation is still controversial. The aim of the present meta-analysis is to evaluate if surgical treatment is more effective in the prevention of recurrent patellar instability than conservative treatment.
**Materials and methods** Studies were identified by searching electronic databases: MEDLINE, EMBASE, Cochrane Central Register of Controlled Trials and CINHAL from 1948 to February 2012. All randomized controlled clinical trials (RCT) and observational studies which compare surgical versus conservative treatment after first patellar dislocation were included. Patients exclusively treated by fixation of osteochondral fractures were excluded. Primary outcomes were: recurrent patellar dislocation, recurrent subluxation, subsequent surgery. We also considered all of the outcomes reported by each study. Statistical analysis of the data was performed by using RevMan 5. RCT and observational studies were analysed separately. The methodological quality of the included studies was assessed using the Cochrane Collaboration's "Risk of Bias" tool. Assessment of the overall quality of the body of evidence and strength of recommendation for each outcome across the selected studies was assessed using the GRADE system.
**Results** 11 studies were included in the meta-analysis: 7 RCT and 4 observational studies. In the RCT the mean follow-up ranged from 2 to 14 years and the mean age was between 12 and 26 years; in the observational studies, the mean follow-up varied between 6.1 and 8.1 years and the mean age was between 9 and 22 years. Conservative treatment was similar in all of the selected studies: immobilization in a brace, followed by an early rehabilitation program. Surgical treatment included many different techniques. The methodological quality of the eligible trials was very limited. There were no significant differences between surgical and conservative treatment for any of the selected outcome measurements.
**Discussion** The poor methodological quality of the selected studies, the small number of patients assessed by each study and the wide range of surgical treatments, do not allow this meta-analysis to provide a clear indication of the choice of the most effective treatment after acute patellar dislocation. Further RCTs with larger sample sizes are needed.
**Conclusions** Based on the results of the present meta-analysis, there are no differences between surgical and conservative treatment after acute patellar dislocation in the prevention of recurrent patellar instability.

### Scapho-lunate injury: about a rational treatment

P. Ghiggio*, R. Lombardo, G. Nobile, M. Pettiti, L. Trifilio

ASLTO4 (Ivrea, IT)

**Introduction** A scapho-lunate ligament lesion is the first step of a carpal instability. The most frequent evolution is a DISI caused by the involvement of the scapho-capitate and scapho-trapeziumtrapezoidal ligament: a scaphoid rotational instability may occur as final consequence. A SLAC may happen in advanced cases. Our purpose is to lay stress on lesion's early diagnosis and treatment.
**Materials and methods** The evaluation was performed on a thirthy-patient series: fourteen were treated within 3 weeks and sixteen were chronic lesions. In recent injuries we repaired the ligament with a temporary stabilization with Kierschner wires completed using two mini anchors. In two cases we used a Herbert screw. A cast for 8 weeks was performed in all cases. A good recovery was obtained in ten cases. Two had a SLAC evolution. Chronic lesions are classified in static and dynamic. Our series included six dynamic (grade 1 and 2) and ten static. The dynamic ones underwent to a dorsal capsulodesis; the static ones had been treated with Brunelli's modified Garcia Elias technique in some cases and the other with ECR sleep.
**Results** The early treatment results are very encouraging: we recurred to a ligament reconstruction in two cases only. Static instabilities had a good percent of results following the Mayo Wrist Score: pain had a significant decrease; strengtness and functional range of motion was reached in 70 % of cases. The instability relapsed in three cases only.
**Discussion** This type of lesions, if not early treated, could evolve to a complicate instability: it must be always suspected in every thraumatisms in extended wrist position. A four-projection X-ray study must be performed as soon and, if not demonstrative, it must be followed by another one with clunched fist. A NMR may be useful to be done after a period of immobilisation.
**Conclusions** The early approach of this lesion is basic to avoid consequent serious damage.

### Lisfranc injuries in sport

U. Alfieri Montrasio*[1], M. Grassi[2], M. Boga[1], M. Easley[3], F.G. Usuelli[1]

[1]CTS, Piede e Caviglia, IRCCS Galeazzi (Milan, IT);
[2]Università degli Studi di Milano (Milan, IT);
[3]Duke University (Durham, N.C., USA)

**Introduction** The incidence of Lisfranc trauma is very low: 0.2 % of all fractures. They are typically the result of high energy trauma (accidents) and 58 % are associated with other injuries. Low-energy Lisfranc injuries are related to sport. In fact, Lisfranc injuries are the second most common injuries of the foot related to sport (second only to phalangeal joint injuries). Most cases are caused by indirect trauma the so called "twisting". These lesions are most commonly described in rugby, football and to a lesser extent in football and in artistic gymnastics.
**Materials and methods** In this study were recruited all patients relating to foot Surgery sports at Duke University (NC-USA) in the academic year 2008–2009 (mostly basketball players and football) for a total of 40 patients. X-ray data bilateral weight bearing and oblique view were collected and divided into 3 groups of classification of Nunley and Vertullo for trauma of Lisfranc low energy. Group 1: trauma of Lisfranc without diastase M1–M2. Group 2: trauma of Lisfranc with diastase M1–M2: percutaneous surgical indication. Group 3: trauma of Lisfranc with diastase M1–M2 and loss of longitudinal arch: open surgical indication. Follow up to 6 months after surgical treatment-bloodless.
**Results** The resumption of competitive sport was granted to 3 months of trauma patients in Group 1 and 6 months for groups 2 and 3. Two patients of Group 3 are not returned to the previous task to trauma.
**Discussion** The trauma of low energy Lisfranc is not a pathology of little importance to the professional athlete, but can represent an obstacle to a return to competitive sport. Therefore, should be treated according to a rational therapeutic algorithm that guarantees reliable and reproducible results in the medium term.
**Conclusions** In the study of trauma of Lisfranc is Rx weight bearing foot associated with bilateral oblique view. It is a major trauma for the athletes who, in 20 % of cases, are diagnosed in the first instance: it is essential, therefore, a high level of suspicion and proper investigation of the imaging diagnosis and proper treatment.

### Reconstruction of the coracoclavicular ligament using an autologus semitedinosus tendon in the treatment acute acromioclavicular dislocation

C. De Ieso*[1], M. Saccomanno[1], L. Deriu[1], G. Logroscino[1], A. Grasso[2], G. Milano[1], C. Fabbriciani[1]

[1]Istituto di Clinica Ortopedica, Università Cattolica del Sacro Cuore (Rome, IT);
[2]Casa di Cura Villa Valeria (Rome, IT)

**Introduction** Acromioclavicular dislocations are classified into 6 types, according to Rockwood. Conservative treatment is indicated in type I and type II injuries; surgical treatment is recommended for type IV to VI; the treatment of acute type III injuries is still controversial. Numerous surgical techniques have been described. This paper describes an anatomical reconstruction of the coracoclavicular ligaments using an autologous semitendinosus tendon in the treatment of acute acromioclavicular dislocations.
**Materials and methods** 18 patients affected by acute type III to V injuries were surgically treated between 2005 and 2009. Autologous semitendinosus tendon was harvested from the knee before acromioclavicular joint exposure. Then, we performed a perpendicular skin incision on the shoulder, distal clavicle excision and drilled a hole where the conoid ligament is attached. The semitendinosus graft was passed through the clavicular hole and underneath the coracoid process. The clavicle was reduced to the anatomical position and the semitendinosus graft was sutured inside the distal clavicle with transosseus sutures. After surgery, a Kenny-Howard sling was applied to the operated limb and was maintained for 6 weeks. After this period, all patients underwent the same rehabilitation program. Primary outcome was recurrent subluxation 6 months after surgery, assessed by an X-ray examination. Secondary outcomes were assessment of disability-related quality of life using the DASH score and the evaluation of global functional status of the operated shoulder using the normalized Constant score.
**Results** 17 male and 1 female were enlisted for the present study. Mean age was 27 years (range: 21–47). Mean follow-up was of 28 months (range: 18–49). None of the patients were lost at follow-up. X-ray examination showed recurrent subluxation in 2 cases (11 %). Mean DASH score was 6 points; mean normalized Constant score was 92 points.
**Discussion** Anatomical reconstruction of coracoclavicular ligaments using an autologous semitendinosus tendon should be considered a reliable technique in the treatment of acute acromioclavicular dislocation.
**Conclusions** Anatomical reconstruction of coracoclavicular ligaments using an autologous semitendinosus tendon showed excellent clinical results at a long-term follow-up.

### Up to date in the diagnosis and treatment of partial ACL tears

S. Cerciello*, M. Vasso, K. Corona, A. Schiavone Panni

Università degli Studi del Molise (Campobasso, IT)

**Introduction** Recently great interest has roused concerning partial ACL tears. This was mainly due to the concepts of double bundles anatomy and biomechanics and the update of double bundle reconstruction technique. The possibility of preserving healthy bounlde in case of partial ACL tears joins the concepts of tissue sparing and minimal impact of surgical procedures. However this issue is still debated with some controversial aspects.
**Materials and methods** In order to clarify this issue a literature review through Medline research was performed. Key words were: ACL and partial tear.

**Results** 200 abstracts were available. 80 were analyzed and 20 full text articles were then evaluated.
**Discussion** Data analysis revealed no consensus on the diagnosis and treatment. Patient history is often similar to the one of complete lesions. Clinical evaluation is sometimes not enough precise to differentiate partial and complete tears. Moreover in some cases, even MRI and arthroscopy are not completely decisive for a correct diagnosis. Evaluation under anaesthesia showing asymmetrical Lachman test and negative pivot shift is the safest tool for a diagnosis of partial tear. In low demanding patients conservative treatment is strongly indicated. In high demanding sportsmen aggressive approach with primary reconstruction or augmentation may be proposed. Some authors suggest to sacrifice the healthy bundle and to perform a single or double bundle reconstruction. The majority stress the biomechanical role of the ACL remnants thus advising the reconstruction of the damaged bundle alone. New trends suggesting the possibility of ligament healing after intra articular injection of growth factor seem promising although no data has been already published.
**Conclusions** Partial ACL tears are more frequent than previously expected in common practice. Precise patient evaluation is crucial since the diagnosis is often difficult. Once the diagnosis is confirmed, proper therapeutic protocol must be followed. This must be adapted to the pathology which is completely different from complete tears.

### *C44–FOOT AND ANKLE 1*

### "One-Step" technique in the treatment of talar osteochondral lesions: results at 4 years and predictability of T2-mapping MRI

M. Cavallo *[1], M. Battaglia[2], R. Buda[1], A. Ruffilli[1], G. Pagliazzi[1], L. Ramponi[1], F. Vannini[1], S. Giannini[1]

[1]II Clinica, Istituto Ortopedico Rizzoli (Bologna, IT);
[2]Istituto Ortopedico Rizzoli (Bologna, IT)

**Introduction** Several techniques have been described to treat osteochondral lesions of the talus. The arthroscopic treatment with autologous bone marrow derived cells (BMDC) using "One Step" technique achieved good clinical and radiological results, overcoming drawbacks of previous methods. Aim of this study was to evaluate the results at 4 years of follow-up in a group of patients treated with "One Step" technique and to correlate clinical outcome with the results obtained from the qualitative analysis of the regenerated cartilage with T2 mapping MRI.
**Materials and methods** From October 2005 to June 2009 49 patients underwent BMDC by arthroscopic surgery in a single step (one step technique). The mean size of the lesions was 2.24 ± 1.23 $cm^2$ (1.50–9.00 $cm^2$), mean depth of 3.9 ± 0.9 mm. Arthroscopic BMDC consists in aspiration and concentration of the bone marrow, loading of the cells concentrated on the scaffold and implantation of the whole biomaterial with arthroscopic procedure and dedicated instrumentation. Patients were evaluated by AOFAS score, X-rays and MRI. T2-mapping MRI was performed at 24 months.
**Results** The preoperative AOFAS score was 63.73 ± 14.13 (26–5), 91.76 ± 7.76 at 24 months follow-up and 82.19 ± 4.17 ($p < 0.0005$) at 46.8 ± 6.8 months. In 20 patients the T2-mapping sequence was performed: in all cases there was a hyaline-like tissue regeneration (T2 35–45 ms-map) extended to 78 % of the regenerated (range 50–92 %). Reassessing T2-mapping data to 24 months with the clinical score, there was a trend for correlation between a high percentage of regenerated hyaline-like type and high clinical score (rho = 0.497, $p = 0.06$).
**Discussion** At follow-up of 24 months the maximum clinical score with excellent AOFAS values has been reached, while there was a modest decline in the clinical score at 36 and 48 months which is then stabilized until the last follow up. It was noticed that a high presence of regenerated tissue with T2 values analogue to the hyaline tissue tends to correlate to the maintenance of a high clinical score at 36 months of follow-up.
**Conclusions** The BMDC with "One Step" technique is able to provide satisfactory results in the mid-term, making possible the regeneration of a tissue with characteristics similar to the hyaline cartilage. The T2-mapping sequence showed to be able to predict the mid-term clinical evolution of a lesion treated with the one-step technique and bone marrow derived cells.

### Achilles tendon and plantar fascia in recently diagnosed type II diabetes: role of body mass index

M. Abate*[1], C. Schiavone[1], A. Pantalone[2], D. Vanni[2], A. Barbati[2], A. Puzzo[2], V. Salini[2]

[1]Dipartimento di Ecografia Internistica, Università "G. d'Annunzio" (Chieti, IT);
[2]Clinica Ortopedica e Traumatologica, Università "G. d'Annunzio" (Chieti, IT)

**Introduction** Previous research has shown that plantar fascia and Achilles tendon thickness is increased in diabetes. Aim of present study was to assess whether tendon changes can occur in the early stages of the disease, and to evaluate at which extent are influenced by body mass index (BMI).
**Materials and methods** The study population included 51 recent onset type II diabetic subjects, free from diabetic complications, divided, according to BMI, in three groups (normal weight, overweight and obese). Eighteen non-diabetic, normal weigth, subjects served as controls. Plantar fascia and Achilles tendon thickness was measured by means of sonography.
**Results** The groups were well-balanced for age and sex. In all diabetic subjects, compared with controls, plantar fascia and Achilles tendon thickness was increased ($p < 0.001$, $p = 0.01$, $p = 0.006$, respectively). A significant relationship was found between plantar fascia thickness and BMI values (r = 0.749, $p < 0.001$), while the correlation between BMI and Achilles tendon was weaker (r = 0.399, $p = 0.004$).
**Discussion** This study shows that plantar fascia and Achilles tendon thickness is increased in the early stages of type II diabetes and that BMI is related more to plantar fascia than Achilles tendon thickness.
**Conclusions** Further longitudinal studies are needed to evaluate whether these early changes can overload the metatarsal heads and increase the stress transmitted to plantar soft tissues, so representing an additional risk factor for foot ulcers development.

### Arthroscopic treatment of ankle anterior bony impingement: a long-term clinical outcome and new classification

R. Buda*, A. Parma, F. Vannini, A. Ruffilli, M. Cavallo, E. Ferranti Calderoni, S. Giannini

II Clinica, Istituto Ortopedico Rizzoli (Bologna, IT)

**Introduction** The arthroscopic treatment of anterior bony impingement of ankle provides initial good results, which tend to decrease over time. Aim of this study was to analyze the statistically significant factors affecting long-term results and propose a new classification system.

**Materials and methods** 80 consecutive patients with a mean age of 37.3 years were treated between 2000 and 2004. Impingement lesions were identified according to Scranton-McDermott classification and, as regards the degree of joint degeneration, according to the classification of van Dijk. In addition, patients were evaluated according to two new specific classification systems, considering location and size of osteophytes and severity of cartilage damage. Patients were evaluated after 24 and 104.6 (mean) months follow-up, following the AOFAS scale. The incidence of the various factors on the outcomes was statistically analyzed.

**Results** The mean pre-operative AOFAS score was 50.9, while at follow-up it scored 70.7 ($p < 0.05$). The Scranton-McDermott classification did not affect the results but only the preoperative stage. The van Dijk scale significantly affected the result but not the preoperative stage. The new classification system proposed has proved to be statistically significant. Tibial localized spurs had better recovery at follow-up. The grade of the chondral lesions affected the outcome. Other factors negatively affecting the results over time were increasing age, cavus foot morphology and previous ankle fracture.

**Discussion** Arthroscopic treatment of anterior bony impingement of ankle provides good overall results, however, the long-term presence of contemporary associated conditions such as chondral lesions, age, previous trauma contributes to the deterioration of the results.

**Conclusions** Based on these assessments is proposed a new classification with prognostic significance.

### Austin osteotomy: comparison of two different fixation methods

G. Riva*[1], T. Binda[1], M. Rossi[2], F. D'angelo[1], C. Montoli[1], P. Cherubino[1], C. Ratti[1]

[1]Dipartimento di Scienze Chirurgiche Ricostruttive e Tecnologie Avanzate (Varese, IT);
[2]Dipartirmento Ortopedia e Traumatologia (Saronno, IT)

**Introduction** Hallux valgus is a very common foot deformity, whose correction has been described in more than one hundred surgical techniques. The aim of this prospective, randomized study is the comparison and analysis of the outcome of two different fixation methods used for fixation of the Austin Osteotomy.

**Materials and methods** Between June 2009 and January 2010, 30 patients were selected based on specific criteria (younger than 60 years, intermetatarsal angle less than 16°, pain related to valgus deformity, full range of motion and reducibility of the deformity, no previous surgery and no other associated deformity). The patients were randomized into two homogeneous groups the group treated A with K-wire (1.8 mm) and the group B with Acutrak® screw. All patients were evaluated clinically and radiographically at 1, 3 and 6 months after surgery. The clinical evaluation was performed using the Hallux Score of the American Orthopaedic Foot and Ankle Society (AOFAS).

**Results** At 6 months the value was 94.06, respectively for group A and 94.45 for group B ($p > 0.05$).

**Discussion** Clinical outcome at 6 months showed no statistically significant differences. However, probably due to the better stability and the absence of percutaneous K-wires, the group B patients could benefit from an earlier mobilization, getting better results in the controls of the first and third month.

**Conclusions** Both fixation devices proved to be effective. Beside the economic aspects, the surgeon should make his choice based on his/her experience and familiarity with a particular fixing method.

### *C45–TRAUMATOLOGY 6*

### Clinical impact of comorbidities in patients with femoral neck fragility fractures

F. Liuni*, E. Gasbarra, A. Scialdoni, M. Feola, N. Habib, U. Tarantino

Università degli Studi di Roma Tor Vergata (Rome, IT)

**Introduction** Elderly patients with fragility fractures of the femoral neck often have several comorbidities that are part of a more complex syndrome, the 'frailty syndrome', which affects their outcome and increases the risk of mortality. The purpose of this study was to evaluate the weight of comorbidities on the risk of mortality at 10 years in men and women with femoral neck fracture.

**Materials and methods** We evaluated 190 patients over 60 years old, surgically treated for femoral neck fracture from January 2010 to December 2011. Patients were divided into two groups according to sex (131 women and 59 men). For each patient was rated the mortality risk at 10 years with Charlson Comorbidity Index (CCI), the days of waiting from the fracture to the surgery and the number of comorbidities.

**Results** The average number of comorbidities in the group of men was 1.5, 1.35 in women. The difference was not statistically significant. The average CCI in men's group was 44.60, 35.17 in women. The difference between the two values was statistically significant ($p \leq 0.05$). Comorbidities more frequent in men were: hypertension (29 patients, 43 %), diabetes mellitus (16 patients, 23.5 %), chronic renal failure (12 patients, 17.6 %), chronic atrial fibrillation (12 patients; 17.6 %). Comorbidities more frequent in women were: hypertension (28 patients, 46.5 %), diabetes mellitus (38 patients, 29.5 %), chronic renal failure (28 patients, 21.5 %), atrial fibrillation (28 patients; 21.5 %). The average days of waiting in men were 2.85, 2.43 in women. The difference was not statistically significant.

**Discussion** The two groups show on average the same waiting period for surgery and the same number and type of comorbidities. Despite this, the group of men showed a higher mean CCI. This result could be caused by the increased incidence of some specific comorbidities (such as ischemic heart disease or neoplastic diseases) in men that could influence the outcome.

**Conclusions** In elderly patients with femoral neck fracture, comorbidities may further aggravate the clinical picture hesitating in the 'Frailty syndrome'. This study demonstrates that, despite a higher incidence of fractures in women, man is more 'fragile' and with a higher mortality risk for a possible susceptibility to complications and a poorer general condition. The study will be expanded with a radiographic follow-up of these patients, with a correlation between potential alterations of fracture healing and comorbidities in individual patients.

### Basic nail: an innovative nail on the lateral hip fractures

F.A. Basile Rognetta Francesco*[1], A. Foti[2], P. Cavaliere[1]

[1]Giomi Reggio Calabria (Reggio Calabria, IT);
[2]Policlinico Universitario " G. Martino" (Messina, IT)

**Introduction** The increase of the average age of the population has led to a growing interest in the fractures of the proximal femur. Therefore, the intramedullary nails represent the gold standard of this treatment. It provides a method of rapid execution that allows an early mobilization of the patients with early resumption of their daily activity.
**Materials and methods** To improve these characteristics, the author has devised the nail "BASIC NAIL", with innovative features. The nail is full, 9, 10 mm in diameter; length 190.250 mm. The tip is tapered to facilitate sliding on the metaphyseal cortex proximal medialis. It has a single cephalic screw self-drilling and self-tapping; the distal locking hole is oval and it allows a dynamic and static distal locking. The inlet hole of the nail is made up of a hand drill of the same diameter proximal end of the nail. The nail length is 250 mm, it also allows an easy treatment of the subtrochanteric fractures, always having a precise guide for the distal locking.
**Results** Since January 2010, over 250 "BASIC NAIL" nails have been planted. The rehabilitation protocol was very early, loaded with walker on the third day after surgery. There were 4 failures, 2 with the protrusion of the screw, caused by the contributory cause of severe osteoporosis, excessive length of the cephalic screw and severe breakdown of the fracture load given too early, 1 "cut off" happened about 3 months after the fracture apparently consolidated, and 1 for incorrect positioning of the screw. The haemoglobin reduction before and after operation was found to be about 1.2 g as compared with 2 g of patients who were implanted with an intramedullary nail according to a traditional technique and by milling the medullary canal; this difference was evident in the fastest functional recovery, in the minor need of transfusions, but also in the reducing recovery times and thus reducing the overall costs of patient management.
**Discussion** The "BASIC NAIL" is an intramedullary nail innovative for the treatment of lateral fractures of the femur. Easy to use, the operative times are reduced and the installation cost is cheaper, these features make it a more suitable alternative to the nails on the market today. Its features allow a faster recovery of patients' fractures, lower operative blood loss and less stress.
**Conclusions** The "BASIC NAIL" nail may reduce operating times by simplifying the technique, this is to the benefit of both the elderly patient and the young surgeon.

### *C46–HIP 6*

### Survival of cementless dual mobility sockets: ten-year follow-up

R. Philippot[1], M. Stamilla*[2], F. Farizon[1], G. Gresta[1]

[1](Saint Etienne, IT);
[2](Catania, IT)

**Introduction** We report a retrospective series at 10 years follow-up of 100 total hip arthroplasties with a double mobility cup. The purpose of this study is to estimate the survival of this cup in 10 years.
**Materials and methods** The studied series contains 100 total hip arthroplasties, implanted in first intention. Series is homogeneous and continue. The used implants are always the same. A stainless cotyle NOVAE SERF© who is a cup covered with ceramic of alumina, with two short contacts of anchoring and one saw superior of mooring and an holding back insert in polyethylene. A screwed stem type PRO 1 SERF© and a chrome cobalt head of diameter: 22.2 mm. The cox-arthrose represents the main indication of arthroplasty and the average age during the implanting is 59.2 years. The group of the patients was regularly revised clinically and radiologically in the service. We studied the survival of this cupule in 10 years by a method actuarielle by taking as end the point the surgical resumption of the cup for aseptic cause.
**Results** We regret 12 deaths and 1 lost sight during 10-year follow-up. The score of Postel-Merle d'Aubigne was 9.6 pre-op. and 16.7 at 10-year follow-up. We observed 2 aseptic loosening, 2 intra prosthetic dislocations by wear of the retention and an advanced wear; so, after 10 years, the rate of global actuarial survival of this cup is 94.8 %. In this series we noted the absence of episodes of prosthetic instability.
**Discussion** This study shows that this double mobility cup possesses a survival in 10 years comparable to the data of the literature. Double mobility does not seem to influence the quality of the acetabular anchoring. The absence of prosthetic instability in 10 years confirms big stability of the double mobility at short- and long-term. The intra prosthetic dislocation, due to the loss of retention by the polyethylene, is the main limit of this technique, but its incidence (2 % in 10 years) is weak and its treatment simple.
**Conclusions** We recommend the pose of this type of cup in subjects with high risk of post operating instability, but also in a systematic way after 75 years of age because instability is the first cause of later surgical resumption in this age.

### The effectiveness of eco-guided infiltrations with high molecular weight associated with administration of oral chondroprotective supplements in osteoarthritis of the hip

D. Scaturro[1], A. Sanfilippo*[2], A. D'arienzo[2], M. D'arienzo[2], G. Letizia Mauro[1]

[1]U.O.C. di "Riabilitazione", AOUP Paolo Giaccone, Università degli Studi di Palermo (Palermo, IT);
[2]U.O.C. di Ortopedia e Traumatologia, A.O.U.P. Paolo Giaccone, Università degli Studi di Palermo (Palermo, IT)

**Introduction** "Osteoarthritis" does not mean a degenerative articular process only but a pathologic expression of wear, inflammation and immunological imbalance of the joints. The articular microenvironment is represented by interactions between cartilage, synovium and synovial fluid that produce a strong and flexible system able to contrast the induced changes of biomechanical load. Chondroprotection is a valid concept in knowledge and therapeutic approach of osteoarthritis [1]. Intra-articular hyaluronic acid can be considered an important joint protection as a mechanical, antinflammatory and analgesic barrier; it lubricates the joint by interacting with lubricina and modulates the activity of chondrocytes and synoviocyties as well [2]. Glucosamine, chondroitin sulphate and collagen type II (SYSADOA) in oral administration act like chondroprotective factors. The intake (above 90 days) in combination of these substances is advantageous in osteoarthrosis of the hip (group I-II of the K-L's scale) [1]. Our study has the aim to demonstrate the effectiveness of eco-guided infiltrations with high molecular weight associated with administration of oral chondroprotective supplements (glucosamine solfate, chondroitin sulphate, hydrolyzed collagen type II, hyaluronic acid and L carnitine fumarate) in comparison with the only treatment with ecoguided infiltrations with hyaluronic acid in patients suffering from primary osteoarthritis of the hip.
**Materials and methods** From January 2011 to February 2012, in the U.O.C. of Rehabilitation, University of Palermo, 75 patients (25 male, 50 female) with symptomatic osteoarthritis of the hip (group II of K-L's scale) were enrolled. Patients aged from 45 and 68 years (average age: 61).The subjects were divided into two groups, A-B. In group A patients ($n = 39$) were intra-articular infiltrated for 3 times every 45–50 days and contemporaneously administrationed with oral chondroprotective supplement, in group B all the patients ($n = 34$) were treated with only intra-articular infiltrations. The evaluation parameters (VAS and Womac for disability scale) are checked at the time of recruitment (T0), after each intrarticular infiltration (T1, T2, T3) and 3 month later of the last infiltration (T4).

**Results** Both groups showed a progressive improvement of pain (VAS scale) and reduction of Womac scale. During follow-up, 3 months after the last infiltration, the data results statistically better in group A vs. group B.

**Conclusions** The ecoguided infiltrations with high molecular weight integrated with oral chondroprotective supplements in mild-moderate primary osteoarthritis show a synergistic effect, allowing the reduction of the pain and the functionality of the hip. 1. Mechanical injury suppresses autophagy regulators and its pharmacological activation results in chondroprotection.

**References**

1. Caramés B, Taniguchi N, Seino D, Blanco FJ, D'Lima D, Lotz M (2011) Arth Rheum doi:10.1002/art.33444
2. Abate M, Schiavone C, Salini V (2012) Hyaluronic acid in ankle osteoarthritis: why evidence of efficacy is still lacking? Clin Exp Rheum

### The use of Dabigatran Etexilate for primary thromboprophylaxis after hip replacement

C. Ratti*, M. Marano, L. Murena, P. Cherubino

(Varese, IT)

**Introduction** The risk of thromboembolic events for patients who have undergone a total hip arthroplasty is about of 45–70 % without a prophylaxis, for this reason a specific thromboprophylaxis is recommended. The use of low-molecular-weight heparins is often characterized by a poor compliance with reference to a subcutaneous administration. The dabigatran etexilate is a direct thrombin inhibitor administered by mouth. The objective of this research is to test a selected population of patients subjected to a primary prophylaxis by Dabigatran Etexilate after total hip arthroplasty.

**Materials and methods** Since November 2010 to December 2011 we carried out a primary thromboembolic prophylaxis by Dabigatran Etexilate with 80 patients who underwent a first total hip arthroplasty. We underwent a retrospective analysis evaluating all the medical records, the outpatient controls after 1–3 months from the operation and a final phone interview. We left off the patients with a moderate or heavy kidney insufficiency, liver insufficiency, coagulation alterations, uncontrolled hypertension, acute ischemic stroke in the previous 6 months, cases of hemorrhagic stroke in the previous 6 months, gastrointestinal or urogenital bleedings. We also excluded patients subjected to a therapy with oral anticoagulants, antiaggregants (except ASA). The dosage was of 220 mg daily for patients under 75 and 150 mg daily for patients over 75, prophylaxis was continuated for 35 days. Collected data: length of the operation, blood loss, haemocrome pre-op. and V gg post-op., surgical drain, blood transfusion, length of the immobilization, post-surgical haematomas, symptomatic Deep Vein Thrombosis appearance, PE major-minor bleedings, adverse events, satisfaction, compliance.

**Results** No events of PE, of mortality correlated to thromboembolic events, one case of Deep Vein Thrombosis, two cases of major bleeding, no major adverse reactions. All the patients, except the patients with DVT, completed the therapy.

**Discussion** The Dabigatran Etexilate has shown its efficacy and safety, not lower than what we learn in literature, for the LMWHs. The compliance and satisfaction for the oral therapy have been higher than what is reported in literature as to a subcutaneous consumption of LMWHs.

**Conclusions** By a retrospective analysis of the selected patients the Dabigatran Etexilate will be an effective alternative to the use of LMWHs for both its efficacy and safety. The examined patients have shown a higher compliance and pleasure for the oral therapy as to a hypothetical subcutaneous one.

### Early dislocation of THA: a multivariate analysis of risk factors

M. Surace*, A. Fagetti, A. Bertagnon, L. Monestier, P. Cherubino

Clinica Ortopedica e Traumatologica, Dipartimento di Biologia e Scienze della Vita (DBSV), Università degli Studi dell'Insubria (Varese, IT)

**Introduction** The early dislocation of THA is one of the most feared complications with an incidence reported in literature from 2 to 5 %. This event is related to several risk factors related both to the patient and to the surgery and to prosthetic design. The aim of our study is to analyze these risk factors and their influence in dislocation.

**Materials and methods** We analized a total of 387 primary THA in 375 patients performed between September 2005 to Dicember 2008 at our institute with femoral head size of 28 and 32 mm and two types of cups, TMT and Trilogy, all were implanted by the posterolateral approach. All patients except 53 had coxarthrosis as preoperative diagnosis. We analyzed sex, age, biometric index and BMI as factors related to the patient. For clinical evaluation we used the Harris Hip Score. The measurement of the femoral offset, abduction and anteversion angle of the cup were realized by radiographyic evaluation.

**Results** We have had 6 dislocations (1.86 %); half of these happened to patients with preoperative diagnosis of subcapital fracture ($p = 0.0271$). We didn't obtain statistically significant results for all the other risk factor analyzed.

**Discussion** The dislocation incidence in our study is in line with literature, like the major frequency of dislocation in the patients with diagnosis of subcapital fracture, which is determined by the greater ROM in the pre-operative period; this result is supported also by the highest result in the Harris Hip Score. The offset was restored in all patients. Abduction and anteversion of the cup were maintained in the "safe range" reported in literature. We obtained a major event of dislocation in the prosthesis with 28 mm head size, but this result wasn't statistically significant.

**Conclusions** The subcapital fracture resulted as a condition that could predispose to dislocation of THA. The influence of the femoral head size, with only 4 mm of difference between the two groups, doesn't seem a condition that influences the incidence of dislocation.

### *C47–BASIC SCIENCE 2*

### Intra-tendinous therapy with PRP for Achilles tendinopathy: color Doppler vascular evaluation

A. Crescibene*[2], J. Almolla[1], M. Napolitano[3], H. Almolla[1], E. Costabile[2]

[1]U.O.C. di Radiologia, A.O. di Cosenza (Cosenza, IT);
[2]U.O.C. di Ortopedia e Traumatologia, A.O. di Cosenza (Cosenza, IT);
[3]U.O.C. di Immunoematologia e Medicina Trasfusionale, A.O. di Cosenza (Cosenza, IT)

**Introduction** Achilles tendinopathy is usually associated with neovascularization; color Doppler consents the evaluation of tissue vascularization, the scope of this study is to search for a correlation between neovascularization and intra-tendinous therapy with PRP.

**Materials and methods** Five patients with elevated rate of sportive non agonistic activity were selected, they presented structural degenerative derangement on ultrasound examination; they were treated with four weekly infiltration with PRP delivered with ultrasound guidance. A color power Doppler evaluation was done at onset of infiltration procedures and a month afterwards, number and site of the newly formed vessels were registered.

**Results** Four out of 5 patients had reduction of the number of vessels, one patient showed increase of 2 in the number of vessels as to those found at base line exam. Three patients showed extra tendinous distribution of the vessels in a ventral position and an intra tendinous distribution for as much as 50 % of the tendon thickness; one patient had ventral extra tendinous vessels and one patient with exclusively intra tendinous distribution. After 1 month time from the last infiltration 2 patients presented exclusively ventral intra tendinous vessels, 2 patients presented peripheral extra tendinous vessels and 1 patient with vessel distribution unchanged .

**Discussion** Tendons heal very slowly, this is due to poor vascularization that hinders the reparation process and results ultimately in chronic tendinopathy. Platelets play the part of physiologic starters and modulators of the healing process. The rationale of the technique lies in the possibility to have a local concentration of growth factors that starts, optimizes and amplifies the process.

**Conclusions** Even though the numbers of cases is small, we registered a reduction in the number of vessels at 1-month controls after the end of procedures in all patients except one, furthermore we documented reduced intra tendinous distribution. The study may provide a guide line for the power colour Doppler ultrasound evaluation of Achilles tendinopathy treated with platelet rich plasma infiltration in highly active sports individuals.

### First in vivo study on transosseus wires tensioning as a mechanical factor affecting the fracture healing process in patients treated with Truelok circular external fixator

A. Peccati*[2], A. Kirienko[1], N. Portinaro[2]

[1]Istituto Clinico Humanitas (Rozzano, Milan, IT);
[2]Clinica Ortopedica dell'Università degli Studi di Milano (Milan, IT)

**Introduction** Mechanical environment is one of the factors affecting the fracture healing process. External fixation is getting more widely used thanks to the possibility to regulate the system stiffness, changing some parameters, such as the transosseus wires tensioning: orthopeadic surgeons can determine stiffness by applying different tensions to Kirschner wires. Many authors proved with in vitro works a wire tension loss during weight bearing in patients treated with external fixator; our aim is to measure this tension loss in patients treated with Truelok external fixator by setting up a specific in vivo pilot study.

**Materials and methods** Between September and December 2010 we treated 12 patients (7 males, 5 female; mean age: 32.7 years) by applying external fixators to 3 femurs, 12 legs, 10 feet. At the surgery time every Kirschner wire has been tensioned to 130 kg. For each patient we chose as reference wire (and measured its tension at each clinical control during follow-up) the one who had shown the least tension loss at the early post-operative control.

**Results** The mean treatment time has been 28 weeks. Each patient has meanly undergone 6 clinical controls (meanly 16 days apart from each other). The mean post-operative wire tension was 117.98 kg, while at the end of treatment it was 32.77 kg (−72.22 %); Kirschner wires tension trend was constantly decreasing for all the follow-up, except for an increase during post-operative week no 9. At the end of the study we calculated the trend-line equation of the wire tension: $y = -3.5621x + 114.59$ ($R = 0.91$; $R^2 = 0.82$).

**Discussion** The negative slope of the trend-line (−3.56) shows an actual Kirschner wire tension loss, and the Pearson's correlation coefficient an excellent linearity of our results and a good correlation with the calculated trend-line. The measured tension loss is smaller than one reported by other Authors, but in our study we introduced too many variables to compare their works with ours and get a statistically solid result.

**Conclusions** In our study we found an actual wire tension loss, as demonstrated by measured values, negative slope of the trend-line equation and Pearson's coefficient. This loss was lower than the one reported by other authors (−78.22 vs. −83.33 %); we can hypothesize Truelok external fixator can offer better biomechanics performances (greater stiffness and less inflammation due to osteolysis at the bone-wire interface). We can conclude that further studies are necessary to confirm this difference from a statistically valid point of view.

### Optokinetic analysis of gait cycle during walking with 1 cm- and 2 cm-high heel lifts

R. Valentini*, M. Toffano, S. Mezzarobba, G. Fancellu

Clinica Ortopedica e Traumatologica, Università degli Studi di Trieste (Trieste, IT)

**Introduction** The use of orthotic devices to raise the heel is available in many cases of degenerative disease of the Achilles tendon as a first step in the conservative treatment. The use of footwear with the heel induces a plantarflexion of the ankle with a resulting decrease of the tension forces acting on the triceps surae. The question is how high must the brace be placed under the heel?

**Materials and methods** We analyzed the gait with optokinetic technique (infrared cameras and reflective markers and baropodometric platform) using two different measures of orthosis under the heel of 1 cm and 2 cm. Each measurement included kinematic and kinetic data with moments of force and angular variations at the level of the hip, of the knee and of the foot. The study included 14 subjects (5 males and 9 females) aged between 20 and 35 years.

**Results** The results of the data arise from the curves of the ground forces and showed statistical significance in the ground reaction forces and in the torques of the knee. And it has showed in a significant ($p = 0.0001$) decrease in amplitude of the curve of the forces reaction who is expression of the force produced by all the lower limb in ground reaction forces.

**Discussion** This is an expression of a reduction of the minimum values that suggest a reduction of the level of the energy absorbed at the time of '"heel strike" like as the maximum values reflect the average of the energy generated at the "toe off".

**Conclusions** This might suggest that the reduced energy absorption with the increase of 2 cm could have a protective effect in those muscles that are most interested in this feature of the absorption of the forces during the heel contact to the ground as the triceps surae in the ankle and rectus femoris in the knee.

### *C48–ACUTE CAPSULO-LIGAMENTOUS INJURIES 3*

### Achilles tendon injuries: comparison of different conservative and surgical treatments. A literature review

L. Morino[1], R. Cerlon[1], A.M. Federico[2], A. Vannicola[2], M. Paonessa[1], A. Bistolfi*[1], S. Ferrero[1], G. Massazza[3]

[1]AO CTO M. Adelaide (Turin, IT);
[2]Università degli Studi (Turin, IT);
[3]AO CTO M Adelaide, Università degli Studi (Turin, IT)

**Introduction** The rupture of Achilles tendon is a very common disease with increasing incidence especially during sport activity. There are still a lot of discussions about the best treatment. The two options can be the conservative treatment or the surgical one: in selected patients, conservative treatment achieves excellent results, even if there is a higher risk of relapse. On the contrary, surgery is associated with risk of skin lesions, infections and general post-operative complications. In any case, the primary objective is to restore the normal length and tension of the tendon, and finally to restore the previous functionality as soon as possible. In fact, untreated Achilles lesion can cause significant and chronic loss of function in the affected patients.
**Materials and methods** An analysis of the literature was conducted with keywords: "Achilles tendon lesion", "Achilles tendon injury", "Achilles Tendon/injuries" AND "Achilles tendon/surgery: 157 articles were selected. The results were further selected using the following limits: the last 5 years, English/Italian, human, adults 19 + years, resulting in 97 articles. Of these, 33 were found to be of significant level.
**Results** Conservative therapy is considered a choice to reserve mainly for elderly people without great functional requirements and with a greater anaesthesiology and surgical risks (infection, iatrogenic nerve injury). The risk of re-ruptures is approximately 20 %. Regarding surgical treatment, there are three categories: open suture, percutaneous ligature and minimally invasive technique. Results are superimposable. The open technique has the major complication of infection, whose incidence is reduced by the other techniques. The percutaneous technique and the minimally invasive one have the advantages of a lower stress surgery and a lower cost. In addition, intra-operative infiltration of autologous platelet gel on the tendon and the augmentation with a cross stitch or acellular tissue grafts seems to give promising results.
**Discussion** There is no agreement in the treatment of Achilles tendon lesions, but there are several techniques that lead to a therapeutic success. Since the type of patient and injury are heterogeneous (a young athlete or elderly man), the winning choice is based on modular treatment of the patient and his functional requirements. Also, the postoperative rehabilitation protocol must be considered even if there is no evidence or standardization about rehabilitation.
**Conclusions** The development and identification of guidelines by scientific societies would be useful for the treatment of this pathology.

### MPFL for patellofemoral joint instability

G. Cerulli*[1], G. Placella[2], E. Sebastiani[2], M. Bosisio[3], G. Potalivo[1]

[1]Nicola's Foundation, Let People Move-International Orthopedic and Traumatology Institute (Arezzo, IT);
[2]Clinica Ortopedica e Traumatologica, Università degli Studi (Perugia, IT)

**Introduction** Patellar instability is a common problem in diseases of the knee extensor. Many etiological factors have been described but in the past few years attention has focused on the importance of the medial patellofemoral ligament (MPFL) as the primary medial stabilizer of the femoropatellar joint. Indeed according to recent studies it contributes to 53 % of medial stability with as much as 60 % at a 20° flexion.
**Materials and methods** Histological and anatomical studies were performed at our laboratory in order to confirm these data and to describe the most common anatomical structure of this ligament. The MPFL function was then studied from a mechanical point of view with biomechanical tests. We then described a ligament reconstruction technique using a hamstring graft to achieve the best anatomical and functional MPFL reconstruction.
**Results** The anatomical, histological and biomechanical studies have been essential to accurately describe the MPFL's anatomy enabling us to create a new set of instruments especially for the reconstruction of this ligament. The clinical data collected from the score systems and the biomechanical evaluations are very reassuring despite the short-term follow-up.
**Discussion** In recent years MPFL reconstruction has become of extreme topical interest on the international scene. However, few studies focus on the correct procedure for ligament reconstruction and even less on the anatomical insertion at patella level. The trapezium shape of the MPFL makes reconstruction difficult and while the femoral insertion is well known and described in literature, little is known about the anatomical insertion at the patella making it complicated to reconstruct and restore its correct anatomy and function.
**Conclusions** Nowadays MPFL reconstruction is considered one of the most reliable surgical techniques for treating patellar instability. Our anatomical technique for reconstruction achieves good functional and biomechanical results with mini invasive methods. The short-term results are encouraging and open up possibilities for further clinical and basic research to improve our understanding of what is today the most important patellar stabilizer.

### Role of Platelet Rich Plasma and Platelet Rich Fibrin in anterior knee pain after LCA reconstruction with bone patellar tendon

M. Valeo, M. Giaracuni*, R. Lanzone, M. Gurzì, L. Di Martino, C. Villani

Dipartimento di Scienze dell'Apparato Locomotore, Policlinico Umberto I, Università di Roma La Sapienza (Rome, IT)

**Introduction** Traditional surgery of the anterior cruciate ligament reconstruction with the use of 1/3 of middle part of patellar tendon causes iatrogenic lesion of the patellar tendon in its middle portion. The rehabilitation protocol that we use provides early articular recovery from 0–90° in the first week and from 90–110° during the second week. The aim of this study is to evaluate the anterior knee pain in patients treated intra-operatively with PRP/PRF in the subsequent postoperative days and the possible impact in the patient's rehabilitation.
**Materials and methods** We evaluated the cases of the last 10 years and short-term results, obtained from relative scale VAS of 20 patients treated with ACL with BPTB. Ten of these patients did not receive treatment with PRP/PRF. The other patients received the preparation of autologous blood (PRF) where the tendon was sutured and the two margins of the lesion were infiltrated with PRP. Both types of patients were prescribed brace locked in extension for 25–30 days, full load immediately, passive kinesis with CPM 0–90° for the first 7 days and 90–110° during the second week, isometric gymnastic for quadriceps muscle and cryotherapy.

**Results** Six out of ten patients treated with PRP/PRF had no pain with the knee flexed in the first 7 days after surgery, the remaining four patients had pain for the first 3 days and then spontaneous regression of pain symptoms. Eight out of ten patients not treated with PRP/PRF had suffered anterior knee pain with a delay in the recovery of motion and then regression of symptoms spontaneously in the seventh post-operative day. The other two patients not treated with PRP/PRF did not have anterior knee pain.
**Conclusions** the PRP/PRF procedure is quite easy. Not all patients treated with PRP/PRF had a benefit from their use as not all untreated patients have anterior knee pain. It is certain that in the absence of contraindications to the use of PRP/PRF and its ease of use especially intra-operatively, in our opinion it is worth using them because in 75–80 % of cases has been successful and patients were allowed to continue post-operative rehabilitation.

### *C49–FOOT AND ANKLE 2*

### Long-term comparative results in paediatric pes planovalgus deformity surgical treatment

R. Cerlon[1], A. Bistolfi*[1], M. Cerrato[2], E. La Marmora[2], A. Andreacchio[3], G. Massazza[4], L. Morino[1], M. Paonessa[1]

[1]AO CTO M. Adelaide (Turin, IT);
[2]Università degli Studi (Turin, IT);
[3]OIRM (Turin, IT);
[4]AO CTO M. Adelaide, Università degli Studi (Turin, IT)

**Introduction** Surgical treatment in paediatric progressive pes planovalgus is based on the implant of different endorthesis. In our study we compared clinical and radiographic results in young adults treated in their childhood with different surgical methods.
**Materials and methods** Sixty patients were evaluated, 30 of them treated with Villadot Kalix endosenotarsic endorthesis and 30 treated with STJ (Pisani) esosenotarsic endorthesis, all of them treated in CTO (by the II Orthopaedic Department) or in the Paediatric Hospital "Regina Margherita" in Turin. The results were established by clinical and radiographic parameters. Were involved patients treated in their 6–15 years, but were excluded patients with deformities resulting from neurologic or post-traumatic problems.
**Results** The mean follow-up was 13 years. Clinical and radiographic results didn't show remarkable differences between the different surgical methods. No serious complications occurred to invalidate the final result. In all patients who have removed the endorthesis, no one showed any mark of intolerance to the orthesis in the AP evaluation. No pathologic contracture was observed in any treated foot. Patients treated with STJ (Pisani) endorthesis reported more post-operatory pain and less tolerance compared to the ones with Kalix endorthesis.
**Discussion** Even if the considered methods showed similar results, must be pointed out that Villadot orthesis is easier and faster to locate and less invasive for the surrounding anatomical structures. For the best result, the treatment must be performed when the skeletal structures are enough well-growth but preserve some development skill to recover the physiological and anatomical structure of ligaments and tendons.
**Conclusions** This study shows the effectiveness of both methods based on the results obtained and the facility of implantation techniques, however emphasizing the minor injury of the anatomical structures of Villadot Kalix endosenotarsic orthesis.

### Actual orientation in the treatment of plantar fasciitis and heel pain: evidence based medicine and literature review

L. Morino[1], R. Cerlon[1], G. Gays[2], A. Vannicola[2], M. Paonessa[1], A. Bistolfi*[1], M. Giuseppe[3]

[1]AO CTO M. Adelaide (Turin, IT);
[2]Università degli Studi (Turin, IT);
[3]AO CTO M. Adelaide, Università degli Studi (Turin, IT)

**Introduction** The most common cause of plantar fasciitis is the heel pain which is a symptom with different aetiology and high prevalence; so comparing the various techniques of conservative treatment becomes really interesting. The purpose of this review is to identify which techniques have been effective and statistically significant.
**Materials and methods** The research was performed using the following keywords: plantar fasciitis, heel pain, extracorporeal shock wave therapy, stretching, plantar fascia thickness, physical therapy, rehabilitation. We used the following limits: time (10 years), human.
**Results** The scales used for further evaluation of the treatments effectiveness are the VAS pain scale and the FFI (foot function index). The most effective therapies in order to decrease the usual plantar overhead in fasciitis are: use of suitable shoes insoles, the rest and stretching. The plantar fascia specific stretching and the treatment of trigger points are essential to accelerate recovery time. The acute treatment with stretching has revealed better results than the low-energy shock waves while in resistant and chronic fasciitis was demonstrated that the high-energy shock waves can bring significant improvements in symptoms. There is evidence, through the use of diagnostic and therapeutic ultrasounds, that a reduction of the thickness plantar coincides with a painful relief; many studies demonstrate that the injection of corticosteroids reduces thickness, perilesional oedema and consequently the pain statistically significant. The low-dose radiation therapy gave good results in controlling pain in the short and long term. The use of botulinum toxin in analgesic level is statistically significant, while no significant to decrease thickness and oedema. Single studies suggest therapies such as acupuncture, exhaust step and IPST (intracorporeal pneumatic shock therapy) which show subjective improvements but not statistically significant.
**Discussion** Many techniques and methods of rehabilitation seem to give a real benefit to the patient although not statistically significant. There are conflicting results in the short- and long-term, presumably because the exact aetiology of plantar fasciitis and the subjective component of the pain do not allow a proper standardization of results. Frequently there is the simultaneous use of physical or minimally invasive therapy and physiotherapy exercises, therefore it is difficult using this type of review to identify which is actually the best treatment during various stages of plantar fasciitis.
**Conclusions** It is desirable the identification of scientifically validated procedures and standardized guidelines for each type of treatment.

### Three-dimensional matrix-induced autologous chondrocytes implantation for talus osteochondral lesions

B. Magnan*, E. Samaila, M. Bondi, I. Bonetti, G.M. Micheloni, P. Bartolozzi

Policlinico Borgo Roma (Verona, IT)

**Introduction** Surgical treatment of osteochondral lesions (OCD) of the talus is indicated at the failure of conservative treatments or in type 3 and 4 according to Outerbridge classification. We can consider MACI technique (Matrix—induced Autologous Chondrocytes Implantation) as an ACI (Autologous Chondrocytes Implantation) evolution.

**Materials and methods** 30 ankles with a mean lesion size of 2.36 $cm^2$ were treated with MACI technique. We clinically and strumentally evaluated patients at 3, 6 and 12 months and at the final follow-up using American Orthopaedic Foot and Ankle Surgery (A.O.F.A.S.) and Musculoskeletal Outcomes Data Evaluation and Management System (MOCART MRI score). Patients were further evaluated at the final follow up with Coughlin score, Van Dijk scale and recovering time.

**Results** The mean follow up was 45 months. The mean AOFAS score varied from 36.9 to 83.9 at final follow-up. Average of Van Dijk' Scale was 141.1. Coughlin score was excellent in 11 patients, good in 13, fair in 5 and insufficient in 1. MOCART score changed from 6.3 to 3.8.

**Discussion** This matrix appeared easy to handle, conformable to the lesion and suitable to be applied by arthroscopy. No correlation between MRI imaging and clinical results was found. 4 ankles required an arthroscopic second look; in all these cases the biopsy's histological result was fibrocartilage. Our results, however, when compared with those reported in literature with other surgical procedures, show no superiority evidence for MACI technique.

**Conclusions** The MACI technique could be considered for single lesion type 3 or 4 according to Outerbridge classification, size more than 1.5 $cm^2$, patients under the age of 50 years and without arthritic degeneration.

### Percutaneous distal lesser metatarsal osteotomy for treatment of metatarsalgia with metatarsophalangeal instability

B. Magnan*, E. Samaila, M. Bondi, I. Bonetti, G.M. Micheloni, P. Bartolozzi

Policlinico Borgo Roma (Verona, IT)

**Introduction** Surgical treatment in biomechanical metatarsalgia is indicated when conservative and orthotic treatment failed. We used a distal lesser metatarsal osteotomy performed by percutaneous technique for the treatment of biomechanical metatarsalgia with metatarsophalangeal instability, without structural deformities of the fingers. The aim of this prospective study was to assess the possibility to achieve with a percutaneous distal lesser metatarsal osteotomy better or comparable results to those reported in the literature with "open" osteotomies, analyzing any advantages or disadvantages in terms of complications, costs, surgical and healing time.

**Materials and methods** 47 percutaneous osteotomies of lateral metatarsal bones have been performed in 29 patients (28 women and 1 man). The patients' average age at the time of the surgical treatment was 60 years. All patients have been clinically evaluated before surgery and at 3, 6 and 12 months after surgery clinically and radiologically with the American Orthopaedic Foot and Ankle Society (A.O.F.A.S.) clinical score.

**Results** Mean follow-up was 18.3 months. AOFAS score changed from 50.03 preoperatively to 84.58 points at 3 months of follow-up, 89.85 points at 6 months and 89.89 at 12 months of follow-up. X-rays showed the consolidation of osteotomy in all cases, with a proper position of the metatarsophalangeal joints. There were no major complications, neither cases of metatarsal head avascular necrosis.

**Discussion** A.O.F.A.S. score showed a significant increase since 3 months after surgery, due to pain remission, with a gradual upward trend at 6 and 12 months after surgery. The low risk of complications can be associated to the mini-invasive nature of this technique, performed in a very short time required for surgery.

**Conclusions** The percutaneous distal lesser metatarsal osteotomy are a good option for the treatment of biomechanical metatarsalgia with metatarsophalangeal instability, in particular in the early stage when conservative and orthotic treatment have failed, sometimes in association with treatment of first ray deformities.

### *C50–TRAUMATOLOGY 7*

### Fat embolism syndrome in polytrauma patients: up to date

G. Rocca*, A. Pallone, M. Spina

USO di Traumatologia, Azienda Ospedaliera Universitaria Integrata di Verona (Verona, IT)

**Introduction** The fat embolism is found in 90 % of long bones single fractures and in 100 % of multiple fractures or during and after hip or knee replacement or knee. Severes ymptoms occur in high energy trauma, in 0.05–3 % of single fractures, up to 10 % after hip arthroplasty and up to 30 % of cases in multiple fractures.

**Materials and methods** The recent review of the literature today recognize to the pathophysiology of FES primarily a mechanical, but also a biochemist reason. Veins and sinusoids in the cancellous bone have their walls adherent to the trabeculae. When a fracture occur, these anatomic relationship helps to keep open vessel lumens allowing many tiny fat droplets (0.8–1.0 $\mu$) to get into the vessels. This explains why the Fat embolism syndrome is most frequently in multiple skeletal lesions than in severe ssoft tissue injury. Since this is a pathophysiological mechanism which originates from venules of small diameter is easily understandable as the primary target organ is represented by the lungs. Nevertheless we describes 3 other mechanisms through which FES may cause damage to other organs.

**Results** The rapid fixation of fractures in polytrauma effectively reduces the onset of ARDS in what representing the best FES prevention. The intramedullary pressure usually rises around a range of 30–50 mmHg. FE occur in the range 50–150 mmHg. Above 150 mmHg, FE incidence can be ten time higher. During reaming or non reaming nailing the medullary pressure can reach values of 650–900 mmHg. Therefore the use of nailing in polytrauma patients with multiple long bone fractures seems to exhibit a high risk of FES.

**Discussion** We present 3 clinical cases analyzing the correct clinical and surgical management in polytrauma patients by reference to most recent literatur evidence and Damage Control Orthopaedic protocol.

**Conclusions** In regarding the literature and the experience gained we suggest the use of Medullary Channel Depletion and Fluted Rods in maintaining low level of intramedullary pressure during long bone osteosynthesis.

### Multidisciplinary approach to complex limb injuries: orthoplastic damage control

G. Monacelli*, S. Lotito, I. Testa, G. Di Laura Frattura, R. Mazzone, P. Braidotti, G. Cassisa, M. Ceretti

Università degli Studi di Roma "Sapienza" (Rome, IT)

**Introduction** The limb complex lesions in high energy injuries are an open challenge to several treatment solutions. Its complexity comes from different expertises' integration.

**Materials and methods** The approach involving damage control strategies is necessary to reduce infective complications and limb amputation.

**Results** The devices of clinical approach entrance with negative pressure medications have completely changed managment approach to injuries.
**Discussion** They are therapeutic strategies of easy execution which have reduced demolitive operations percentage.
**Conclusions** The authors want to signal the particular experience behaviour with external fixation, VAC therapy and wound care in limb injuries management.

### Organization, achievement, and results of a Pelvis Surgery Center

M. Massobrio*, M. Calò, P. Albanese, G. Antonietti, U. Bianco

Dipartimento Scienze dell'Apparato Locomotore,
"Sapienza" Università di Roma (Rome, IT)

**Introduction** Pelvic fractures are not frequent, yet severe injuries, often associated to other lesions. Well defined diagnostic and therapeutic procedures are absent, and their economical assessment is inadequate. The goal of this study is to propose the organization of a multidisciplinary Center that can develop diagnosis, treatment, and follow up protocols.
**Materials and methods** Twenty-five patients were treated from August 2008 to July 2010, 5 women and 20 men, average age 34.5 years. Twenty patients had acetabular fractures (8 posterior wall fractures, 2 anterior column fractures and 10 mixed fractures, Judet and Letournel). Five patients suffered from diastasis symphisis pubis (three patients with a CAP type I, and 2 with a CAP type II, Young–Burgess). More than 50 % of patients had an associated injury. The diagnostic protocol included a clinical and radiological assessment with X-rays, CT scan and/or angiography. The emergency treatments included hemodynamic stabilization, pelvis immobilization and the hip dislocation reduction, if present. In 20 cases it was followed by a definitive surgical operation. Multidisciplinary collaborations were established. Patients' follow up included radiological and clinical evaluations (Harris hip Score). Considering the 20 patients with acetabular fractures, 14 were treated surgically, 5 incruently, 1 with external fixation. In 10 cases an Iselin surgical access was used, in 4 cases both ileo-inguinal and Iselin access (2 surgical steps). In 5 patients with diastasis symphisis pubis external fixation was used.
**Results** Average delay between trauma and operation was 15.6 days. Average hospital stay after surgery was 45 days. Five had excellent results, 15 were good, and 4 presented poor results. One patient deceased. Four patients underwent hip arthroplasty 1 year after the first pelvis surgery.
**Discussion** It was essential to identify the collaborating units. The center aims at a uniform and rapid treatment for patients with lesions which are treated differently depending on the department of hospitalization and on the surgeon's experience. The target is to avoid treatment delays, costs and complications increases. The RAD evaluation grants the highest value to pelvis surgery. This should be followed by dedicated structures that can become reference centers.
**Conclusions** The results can be improved, but considering this is a not well known context both clinically and economically, they can be seen positively.

### Shoulder hemiarthroplasty in complex fractures of proximal humerus

E. Siuni*, I. Pusceddu, B. Orrù, V. Pace, G. Sulcis

Servizio Chirurgia Spalla, SCOMR Ospedale Marino, ASL Cagliari (Cagliari, IT)

**Introduction** We report our experience in the use of the shoulder prosthesis in recent complex fractures of the proximal humerus, when the comminution and the decomposition of fragment does not allow a stable synthesis and/or the risk of the humeral head necrosis is very high.
**Materials and methods** From March 1996 until now we have tested prosthetic humeral head replacement in 95 patients, 69 females and 26 males, aged between 55 and 84 with an average age of 73 years. In 15 cases, the fracture ad occurred for a high-energy trauma (car accident), whereas in 77 cases for low-energy trauma (accidental fall). 66 patients presentes complex fracture of proximal humerus, whereas 29 cases were of fracture-dislocations. In 12 patients there were also other associated fractures.
**Results** 2 patients with dislocation of humeral head at the time of the surgery showed clinical signs of brachial plexus palsy then regressed during the postoperative period. There was no intraoperative complication, but, in the postoperative period, there was 1 case of myocardial infarction and 1 case of head prosthesis dislocation for an accidental fall 15 days after the surgery. The results obtained, evaluated clinically under the Constant-Score and thank to X-rays with orthogonal projection, have been good or excellent in 61 % of cases and mediocre o bad in the other 39 %. Instead the subjective judgment of the patients was in 69 % satisfied or very satisfied and partially satisfied or dissatisfied in the 31 % of cases.Among the complications emerged the primary or secondary malposition, with the non-consolidation or the resorption of tuberosity, the rupture of rotator cuff with lifts of the humeral head, some periprosthetic calcifications and 2 cases of infection.
**Discussion** Shoulder hemiarthroplasty in complex fractures of the proximal humerus in the elderly patients, has proved a surgery with brilliant clinical results in several cases (especially in patients with a rotator cuff intact), but sometimes also daunting, and further studios have helped to change both the materials used and their design (prosthesis dedicated to fracture), but also the surgical techniques that can be used to improve healing of tuberosity around the stem prosthesis and consequently its functionality.
**Conclusions** The reverse shoulder arthroplasty is more indicated in elderly patients aged over 75 years, with associated shoulder arthropathy, with fragmentation of tuberosity or with rotator cuff tear.

### Shoulder prosthesis in the complex fractures or fractures with dislocation of humeral head: comparison between endoprosthesis and reverse prosthesis

P. Esopi*, G. Novello, M. Dabalà

U.O Ortopedica Traumatologica, ULSS 13 Regione Veneto (Dolo, IT)

**Introduction** Authors apply shoulder's prosthesis in split 4 fragments fractures of humeral proximal extreme, dislocation fractures of the humeral head, compression fractures involving more than 40 % of the articular surface.
**Materials and methods** We analysed shoulder surgery cases of Dolo's Orthopaedic Unit from January 2009 to December 2011. 35 shoulder prosthetic implants were applied in patients with fractures of the proximal humeral epiphysis: reverse-geometry prostheses in 62 % of cases and in 38 % of cases endoprostheses. Average age was 73 years (57 min–93 max). On the left side of the prevalence (57 %) and females (80 %), rating by the score of Constant follow-up period of 17 months.
**Results** 73 pts. mean Constant score for reverse-geometry prosthesis and 65 pts. for endoprosthesis. Pain (15 pts. max): 14 pts. for reverse-geometry prosthesis and 13 pts. for endoprosthesis Activities of Daily

Living (20 pts. max): 18 pts. for reverse-geometry prostheses and 16 pts. for endoprosthesis. Range of motion: internal rotation limitation in all patients. Best functional results were obtained in reverse-geometry implants. Complications: 1 case of dislocation, 2 cases of stiffness, 2 cases of heterotopic calcifications in endoprostheses, 1 case of iatrogenic fracture of the diaphysis and 1 case of infection in reverse prostheses. Detection of torque strength of operated limb in abduction at 90° to the contralateral: all patients maintained at least 50 % and many of them > 75 % of strength in comparison with non-operated limb.

**Discussion** In our experience fractures of the proximal humeral epiphysis in which there are signs for the application of a shoulder prosthesis: (1) it is not convenient procrastinate the application trying osteosynthesis, the subsequent intervention for removal the fixation then applying the prosthesis will be more complex and laborious with worse functional outcomes; (2) reverse prosthesis gives the functional results far better than the endoprosthesis; (3) several factors can affect the functional outcome of endoprosthesis like nonunion, migration and resorption of the tuberosities even if well performed in systems.

**Conclusions** In our series: (1) the functional requirements of the patients had been met; (2) we applied much more reverse-geometry prostheses with better results; (3) we found more difficulty applying protesthesis in results of fracture; (4) we did not found problems of Scapular notch.

### *C51–PAINFUL PROSTHESES 4*

### Painful tibiotarsic prosthesis: diagnostic algorithm

M. Massobrio*, G. Corsi, G. Antonietti, P. Albanese

Dipartimento Scienze Apparato Locomotore, “Sapienza” Università di Roma (Rome, IT)

**Introduction** The success of ankle prosthesis is influenced by 2 factors: the range of motion restoration and the pain disappearance. The range of motion improvement can be partial without compromisig the result, but the absence of pain is the primary target. The aetiology of pain in a patient with a painful tibiotarsic prosthesis requires the analysis of the operated joint, of the ones nearby, and of the indication, execution and management criteria of the prosthesis.

**Materials and methods** We investigate the pain location and the onset time. Location: it allows differentiating the prosthetic or primitive causes from the non-prosthetic or secondary causes. Pain onset time: it appears in the immediate postoperative time and at the resumption of weight bearing in the cases of wrong surgical indication, prosthetic size, relation, alignment, and fractures. It shows after 6 months in case of prosthetic mobilization, tendinous retraction or impingement. The diagnosis requires the execution of a comparative X-rays in the weight bearing situation, of a CT scan (arthritis, bone stock, periprosthetic osteolysis, prosthetic relations) and must be anticipated by a clinical examination and by the clinical analyses of the ambulation and without weight bearing.

**Results** We applied the etiological, topographic, and temporal analyses of pain to 20 patients operated on with tibiotarsic prosthesis from 2008 to 2011. In 2 cases, the pain cause was a subtalar and scaphoid talar joints arthritis. In 3 cases it was due to Achilles and peroneus tendons retraction. In 2 cases it was an oversizing of the talar component, in 1 case due to an intra-operative malleolar fracture. In 2 cases a surgical revision or an Achilles tendon lengthening was performed. In no case a secondary prosthetic mobilization occurred. The subtalar and scaphoid talar joints arthritis were not treated surgically with arthrodesis.

**Discussion** The pain causes in tibiotalar prosthesis can be preoperative, intraoperative and postoperative. Preoperative causes: prosthesis with subtalar and/or scaphoidtalar joints arthritis, tibiotarsic instability, valgus or varus deformity of more than 5°–10°, severe preoperative range of motion reduction, insufficient bone stock. Intraoperative causes: prosthetic malalignment, incongruous prosthesis size, fractures. Postoperative causes: mobilization of prosthetic components (septic/aseptic), tibiotarsic subsidence and impingement, tendinous retraction (Achilles and/or peroneus tendons).

**Conclusions** The diagnostic accuracy of pain causes in tibiotarsic prosthesis is essential because the prosthetic causes can require the implant sacrifice and arthrodesis, while the secondary causes can be surgical, but lighter.

### Pain in total knee arthroplasty: causes and resolutions

E. Gasbarra*, C. Rao, M. Celi, N. Cuozzo, P. Pistillo, R. Iundusi, U. Tarantino

Università degli Studi di Roma Tor Vergata (Rome, IT)

**Introduction** Knee replacement surgery is one of the most effective in the resolution of pain and in the restoration of function in an arthritic knee, with success rates greater than 90 % and a longevity of about 10–15 years. The growing number of primary arthroplasties, with over 55,000 implants performed in Italy in 2008, and increased life expectancy of patients led to an increase in the number of revision surgeries, especially in women and men over-75 with an increase respectively 220 and 300 % in 10 years. Surgical indications include: pain, stiffness or instability of the knee, loosening or failure of osseointegration of the components, or infection, with possible following revision in two stages.

**Materials and methods** Between October 2010 and December 2011 we performed 12 revisions of total knee arthroplasty: 11 patients (10 women and 1 man, of which 1 bilateral) with a mean age of 73.4 years (range 64–80) after a mean interval from primary arthroplasty of 5.4 years (range 2–8). In the remaining case, has been reviewed only polyethylene. Patients were followed with clinical and radiographic controls at 1, 3, 6 and 12 months (mean follow-up 10.9 months). Functional assessment was performed preoperatively and 6 months postoperatively using the Knee Society Clinical Rating System and the SF-36 questionnaire.

**Results** In post-operative recovery was evaluated reestablishment of correct axes of the load and the stability of the knee resulting in restoration of normal walking. The mean Knee Score at 6 months, after the revision, was 75.4 (range 51–94), with a significant improvement over the pre-operative condition (mean 41.5, range 21–65). The scores were excellent (> 85) in 58 % of patients and poor (< 60) in 8 %. The results of the questionnaire SF-36 were strong, with an improvement compared to the condition prior to the revision.

**Discussion** The vast majority of patients show good results in terms of resolution of pain and increased function of the operated limb, resulting in an increase in physical and social activities.

**Conclusions** The surgeon facing a revision knee replacement often has to solve many technical problems, such as malalignment, bone loss or a diminished capacity for osseointegration of the new prosthesis in the presence of decreased bone quality. Revision surgery is, therefore, highly complex; however, most patients are satisfied with the procedure, having had a significant improvement in quality of life.

### *C52–PAEDIATRIC ORTHOPAEDICS 1*

### The effect of anterior cruciate ligament (ACL) lesions on the articular cartilage of growing goats

F. Falciglia, A. Poggiaroni*, M. Giordano, A.G. Aulisa, G. Mastantuoni, V. Guzzanti

Ospedale Pediatrico Bambino Gesù (Rome, IT)

**Introduction** The development of the growing articular cartilage depends on the ability of the tissue to answer to the mechanical and hormonal stimulations. When mechanical forces exceed the physiological limits, irreversible lesions may occur which can modify the integrity of this complex tissue, and lead to arthritis. In the adult, the presence of an ACL lesion leads to an instability of the knee, a predisposing condition to degenerative alterations of the articular cartilage. Anterior cruciate ligament injury in teenagers is today such a frequent event that it receives a great deal of attention. The therapeutic solution is controversial. We do not know for how long we can postpone surgery without causing irreversible damage to the articular cartilage in children with anterior cruciate ligament injury. Up to now, there have been no studies that describe the pathological findings and the evolution of the lesions of the articular cartilage during the growing period.
**Materials and methods** The study was performed on 16 growing male goats of approximately 6 months of age. A complete lesion of ACL was performed with removal of the ligament at arthrotomy. The animals were sacrificed two for group at intervals of 1, 3, 6, 9 months from the operation. The goat knees were submitted to a macroscopic and microscopic evaluation in paraffin and 5 μm sagittal histological sections of the specimens, stained with haematoxylin-eosin, Alcian-P.A.S. and Safranina O. Cartilage changes were evaluated by the Mankin score validated according to Van der Sluijs. Menisci were studied with 5$\mu$ axial histological sections stained with haematoxylin-eosin.
**Results** The progressive deterioration of the morphology and structure of the articular cartilage in the samples begin at 1 month from the operation and complete at 9 month from the operation. The internal menisci show transversal incomplete lesions after the first and at 3 months from surgery and progressive complete lesions at 6 and 9 months post surgery.
**Discussion** The histological observations showed that the complete ACL lesion causes irreversible articular cartilage alterations in growing goats 3 months after injury. Partial ACL lesion does not induce secondary modifications of the growing articular cartilage in goats.
**Conclusions** These experimental data suggest that it would be better to perform ACL reconstruction in growing patients with ACL injury and instability without waiting until the end of growth.

### Osgood-Schlatter lesion: histology of the different stages of the disease

F. Falciglia, G. Mastantuoni*, A. Poggiaroni, M. Giordano, A.G. Aulisa, V. Guzzanti

Ospedale Pediatrico Bambino Gesù (Rome, IT)

**Introduction** From histological, clinical and radiographic observations, various hypotheses have been advanced about the etiopathogenesis of the Osgood-Schlatter (O–S) lesion. Up to now, no study reported the histological features of the various zones of the anterior tubercle of the tibia in the different stages of the O–S lesion. With the aim to assess the characteristics of these zones we carried out a histological evaluation during O–S lesion in patients undergoing surgical treatment in various stages of growth.
**Materials and methods** Specimens were taken from 13 patients with O–S lesion, four in the apophyseal stage and nine in the epiphyseal stage of the anterior tibial tuberosity. Core biopsies were obtained using a Jamshidi needle prior to surgical fixation. Specimens were prepared and stained with hematoxylin and eosin and Masson's trichrome.
**Results** In the apophyseal stage, lesions were present in an altered fibrocartilage anterior to the ossification centre. Reparative tissues were also observed in the upper part of the secondary ossification centre. In the epiphyseal stage, varying degrees of reparative tissues were observed in the bed of the fragment of the secondary ossification centre. In 3 out 9 patients a zone of lesion was observed within the fibrocartilage anterior to the ossification centre.
**Discussion** Our study documented that O–S lesion was present in the apophyseal stage in the fibrocartilage anterior to the secondary ossification centre. The same observation was made in the epiphyseal stage in association with different reparative tissues inside the ossicle and in its bed, suggesting that the slippage of the patellar tendon insertion may be progressive and caused by a pathological fibrocartilage.The various stages of repair described in previous histological studies within the secondary ossification center are to be considered as the result of a lesion occurred in an earlier stage of development, when the cartilage anterior to the secondary ossification centre is not yet ossified.
**Conclusions** Considering the period of growth when O–S lesion more frequently appears, the cause of this cartilage weakness may be similar to that observed in slipped capital femoral epiphyses. This would lead us to consider this disease as a progressive slippage of the patellar tendon insertion within a pathological cartilage anterior to the secondary ossification centre of the anterior tibial tubercle.

### Minimally invasive treatment of distal tibial physeal injuries with cannulated screws

P. Di Giacomo*, G. Federico, A. Troianiello, G. Di Gennaro, R. Ragozzino, A. Borgnino, P. Guida

[1]A.O.R.N., “Santobono Pausilipon Annunziata” (Naples, IT)

**Introduction** Injuries to the distal tibial and fibular physes accounts for 25–38 % of all physeal fractures and are most common between 11 and 15 years of age. These lesions require an accurate articular reduction and good primary stability. Minimally invasive techniques have a primary role in treatment because of large hemarthrosis and the need to respect the physis.
**Materials and methods** In the period 2008–2010 we have treated 141 patients between 11 and 14 years of age. Lesions are classified in 7 types according to Tachdjian and Bias classification. 85 lesions (55.9 %) was supination-eversion-externally rotation lesions (S.E.E.R.); 44 (28.9 %), pronation-eversion-externally rotation lesions (P.E.E.R.); 3 supination-inversion lesions (S.I.); 3 plantar flexion lesions (P.F.); 3 axial compression lesions (A.C.); 1 triplanar fracture and 5 Tillaux fracture. In 84 patients (59.6 %), treatment consisted in reduction and fixation of tibial fracture with Kirschner wires and cast; in 57 patients (40.4 %), in fixation with cannulated screws. This study is focused on MIS treated patients. In these cases a plaster splint or orthosis is applied for 15 days after treatment, followed by range of motion exercises. Weight bearing is allowed at 4–5 weeks. ROM is totally recovered within 4–6 weeks.

**Results** We had good results in 55 cases with complete articular ROM restoration, no length discrepancy or angular deformities and unsactisfactory in 2 patients due to ROM deficit; length discrepancy (1.5 cm.) and angular deformities (5° approximately).
**Discussion** In treating physeal fractures, we must associate articular reduction with respect of the physis and a good primary stability guaranteed by a valid fixation. So a gap between the fracture fragments greater than 4 cm is unacceptable. The use of less invasive fixation devices is also necessary, to reduce the risks of intra-articular penetration and limit the extension of surgical exposure. It is preferable to use partially threaded screws to realize an effective compression of the fracture fragments.
**Conclusions** Distal tibial and fibular physes injuries fixation with cannulated screws is particularly effective allowing achieving primary stability necessary for early mobilization and weight bearing through an adequate compression and reduction of fracture fragments. In this way joint stiffness due to prolonged cast immobilization can be avoided.

### Outcome of the treatment of tibial distal physeal injuries in adolescents

G. Riva*, T. Binda, F. D'Angelo, P. Cherubino, C. Ratti

Dipartimento di Scienze Chirurgiche Ricostruttive e Tecnologie Avanzate, Università dell'Insubria (Varese, IT)

**Introduction** The physeal injuries of the distal tibia are among the most frequent in the adolescent between 10 and 15 years. The risk of epiphysiodesis is important, with morphological and functional consequences often difficult to handle, although not frequent. The prognosis depends on the type of injury, on the trauma energy and on the displacement of the fragments.
**Materials and methods** Between January 2005 and June 2011, excluding patients with type I, II and V Salter Harris lesion, 32 patients underwent surgical treatment at the orthopedic clinic of Varese for physeal injury of the distal tibia. The diagnosis was Salter Harris type III (Tillaux's fracture) injury in 22 patients (71.4 %) and Salter-Harris type IV injury in 10 cases (32 %). All patients were studied by standard Xrays exams and by CT scan for a correct classification of the lesion. All patients showed a more than 2-mm-displacement and underwent surgical treatment. In 26 patients we performed closed reduction and percutaneous fixation; 6 patients required open reduction and internal fixation of the fracture. Patients were assessed clinically at 1, 3, 6 and 12 months with standard X-rays at 1 and 6 months after surgery. Clinical outcomes were evaluated according to the criteria of Burwell and Charnley.
**Results** At a 43-month follow-up (range 9–84) clinical results proved to be excellent. No wound complication occurred in this series of patients. In 17 cases fixation devices have been removed at a mean time of 9.3 months (range 6–15); in other cases it wasn't necessary any other further surgical procedure. There was no significant dysmetria (> 0.5 cm) or disalignment of the ankle compared to the other side.
**Discussion** In this series of patients, there were not statistically significant differences between the groups with various types of treatments available. Our results proved to be concordant with those reported in the literature. Morevover computed tomography proved to be fundamental diagnostic investigation for a more precise understanding of the lesion and for its correct surgical treatment.
**Conclusions** An anatomic reduction, with or without open surgery and internal fixation, is the most important prognostic factor, which can reduce the rate of complications and sequelae.

### *C53–PAINFUL PROSTHESES 5*

### Tibial slope and gaps balance in knee arthroplasty

F. Boniforti*, F. Giacco, A. Macaione, D. Di Marzo

Fondazione San Raffaele Giglio (Cefalù, IT)

**Introduction** The success of total knee arthroplasty relies on the appropriate limb alignment with balanced flexion and extension gaps. Saggital plane cuts are involved and posterior slope require restoring the original.
**Materials and methods** From September 2006 to January 2009, 173 primary CR total knee arthroplasties were performed. Hospital records and radiographs were available for 136 patients (158 knees). The average age at the time of surgery was 69.4 years. The diagnoses included osteoarthritis in 146 knees (95 %). A retrospective review was performed by an independent observer to determine whether a posterior cruciate ligament release had been done; whether a manipulation had been done; and the range of motion noted at 6 weeks and at the time of the most recent follow-up. The posterior tibial slope was measured on X-ray lateral view using a calibrated picture.
**Results** The median tibial slope was 4°. The posterior cruciate ligament was released in 38 % knees. The posterior cruciate ligament was released in 46 % of the knees with < 4° of tibial slope and 29 % where slope > 4°. Manipulation was performed in 5 cases (2 in < 4° and 3 in > 4°), and they not relied on PC release. At the most recent evaluation the ROM was better in the patients with < 4°of posterior slope (115°) than it was in the patients with > 4° of slope (110°). The patients who did not have a posterior cruciate ligament release had similar ranges of motion regardless of the tibial slope.

### Cortical fragment of the lateral femoral condyle transport technique in knee arthrodesis with the Ilizarov method

A. Kirienko*[1], A. Peccati[2], A. Bruno[2], J. Tamini[3], E. Arnaldi[2], N. Portinaro[3]

[1](Milan, IT);
[2]Istituto Clinico Humanitas (Rozzano, Milan, IT);
[3]Clinica Ortopedica dell'Università degli Studi di Milano (Milan, IT)

**Introduction** Knee arthrodesis after TKA is a technique indicated in cases where TKA revision is not possible because of bone loss, extensor apparatus deficiency, great ROM reduction, involvement of soft tissues around the knee or periprosthetic infection. The cortical transport technique (osteotomy of the lateral femoral condyle and its medial translation to fill the bone gap) is indicated in those cases where the patient's age or presence of important osteoporosis can controindicate the bone graft to fill the gap or the proximal and distal bone lengthening because of the small healing possibilities.
**Materials and methods** Between 2008 and 2011 we treated 10 patients with the cortical transport technique (9 females, 1 male; mean age at surgery: 73.34 years; 4 right and 6 left sides). In 3 cases the diagnosis was aseptic mobilization of the prosthesis, and in 7 cases it was periprosthetic infection. 1 patient underwent knee arthrodesis with intramedullary nail, 5 patients underwent prosthesis removal (substituted by antibiotic spacer), while in 4 cases such surgery was performed together with the frame application.

**Results** The mean treatment time was 42 weeks. The mean follow-up time was 32 months. In 5 cases a good clinical, radiographic and functional outcome was gained, in 3 cases it was excellent, while 1 case was complicated by fracture at the arthrodesis site 1 month after the frame removal (the patient was re-operated) and 1 in case pseudoarthrosis at the arthrodesis site was diagnosed. The mean limb shortening reported at the end of the treatment was 2.5 cm.

**Discussion** The results were more than good, and we think we can use this surgical technique also in less severe cases, in order to shorten the treatment time and improve clinical, radiographic and functional outcomes.

**Conclusions** The cortical transport technique, together with the Ilizarov external fixator, allows knee arthrodesis in those patients in whom other techniques would be contraindicated because of the patient's age or clinical conditions.

### Pain in the reverse shoulder arthroplasty

E. Siuni*[1], I. Pusceddu[2], B. Orrù[2], V. Pace[2], A. Mastellone[2]

[1]Servizio Chirurgia Spalla, SCOMR, Ospedale Marino (Cagliari, IT);
[2]SCOMR, Ospedale Marino (Cagliari, IT)

**Introduction** The purpose of this study is to evaluate at a short-medium-term, how many patients undergone surgery for a had reverse shoulder arthroplasty (RSA) may have a significant residual pain and its the possible causes.

**Materials and methods** From 2006 to 2010 we treated 56 reverse arthroplasty surgical patients, 52 women and 4 men, mean age 74 years. 41 if them were operated for a pseudo-paralytic shoulder pain resulting from inveterate massive irreparable rotator cuff tear (6 rheumatoid arthritis); 12 patients were operated due to a disabling sequelae of the complex proximal humeral fractures, from this group 6 had already undergone surgery for osteosynthesis and 3 for hemi-arthroplasty; other 3 were operated for inveterate anterior shoulder dislocation associated with massive rotator cuff tear. All patients were operated by the same surgeon and were evaluated with a follow-up period of 3 years, with Constant-score and recent X-ray examination by other Orthopedics Doctors.

**Results** Nine (16 %) on 56 patients operated with RSA complained a significant pain that, in five cases involved a revision surgery. Going into details, of the 41 patients treated with RSA for massive rotator cuff tear, 3 patients (7.3 %) were subjected to a re-operation, one for infection (debridement and targeted antibiotic therapy for 6 months) and two cases for mechanical failure with instability of glenoid prosthesis and marked bone resorption. From the twelve patients treated with RSA results for fracture, five (41 %) complained pain, 1 for loosening, 3 for residual of the humeral tuberosity displacement and 1 underwent revision surgery RSA in the CTA hemiarthroplasty for buching of glenoid prosthesis and marked glenoid bone resorption. Considering the 3 patients treated with RSA for inveterate shoulder dislocation, 1 complained pain for scapular notch, but it currently does not require surgical treatment.

**Discussion** The clinical and radiographic results, with a short-medium-term follow-up, showed that the RSA in the treatment of the sequelae of fractures proximal humerus has been less brilliant than its use in cuff tear artrophaty.

**Conclusions** The reconnection and the healing of the tuberosity between the two of them and the metaphysis may help to reduce the pain and to improve more and more the functional performance.

### Painful unicompartmental knee prostheses: wrong indication, wrong surgical technique or wrong implant choice?

F. Rodolfo Masera, A. Bassani, M. Rodolfo Masera

Istituto Clinico Città Studi (Milan, IT)

**Introduction** Unicompartmental knee arthroplasty is a valid surgical solution to relieve the knee arthritis. Sometimes however, the result is not satisfying, due to major complications or just because the prosthesis stays "painful".

**Materials and methods** We have evaluated 200 patients, who had undergone a UKA between 2004 and 2010, getting clinics and X-rays exams done.

**Results** The clinic results were satisfying in 90 % of the patients showing a significant improvement of the indexes used. What happened in the remaining 10 % of the cases? Was there a wrong indication? Was this a problem due to a wrong surgical technique or to a wrong choice of the implant?

**Discussion** As concerning the indication: lateral arthritis already affecting the knee, widespread or external patella arthrosis or ACL reduced grip strength, excessive deformity in varus flexed with collateral internal ligament deficiency, rheumatic arthritis active or diagnosed at a later stage. Concerning the surgical technique: sagittal tibia cut: ACL injury, posterior cortical injury, intra and extra rotation with choice of the implant size to follow. Horizontal Tibia cut, excessive or lowed sloop, 90° varus-valgus. Distal femoral cut by means of an endomedullary guide, parallel with the tibia with alignment in extension. Intra-extra and medial–lateral femur positioning. Liner: Varus-valgus final result (tibia-femoral cut, liner). Concerning the implant: femur and tibia component.

**Conclusions** The most important factor is the indication: applying of severe selection criteria, implying not just proper preoperative instrumental screening exams, but also an accurate clinic examination and a detailed medical history of the patient. These are the key factors to ensure a proper selection of the patients and a satisfying final result of the UKA. As well important is the surgical technique, but in this case, the learning curve of this surgical technique, sometimes wrongly supposed to be easy, is the price the surgeon has to share unfortunately with the patient. The choice of the implant is surely less important, even though the implants having more resurfacing on the femur, the mechanism ensuring the primary stability and the choice of a metal back tibia component or of an all poly can surely impact on the results in the short and long term.

### Painful knee: an advanced protocol for the diagnosis of infections. SEM and sonication

R. Ferrari[1], A. Raglio[2], F. Farina[2], G. Gregis[3], S. Buoro[4], G. Virotta[5], A. Remuzzi[6], C. Castelli*[1]

[1]Dept. of Orthopaedics and Traumatology, Ospedali Riuniti di Bergamo (Bergamo, IT);
[2]Dept. of Microbiology, Ospedali Riuniti di Bergamo (Bergamo, IT);
[3]Dept. of Infectious Diseases, Ospedali Riuniti di Bergamo (Bergamo, IT);
[4]Dept. of Clinical Biochemistry Lab., Ospedali Riuniti di Bergamo (Bergamo, IT);
[5]Dept. of Nuclear Medicine, Ospedali Riuniti di Bergamo (Bergamo, IT);
[6]Mario Negri Institute (Bergamo, IT)

**Introduction** Total knee arthroplasty is a successful procedure. Most serious complications have decreased in the past 10 years, but not infection with an incidence reported in the literature stable around 2 % in first implant and between 6 and 40 % in revision surgery despite the use of perioperative antibiotics. A painful knee, early and late knee revision arthroplasty considered aseptic, may be related to a septic context misunderstood. The diagnosis or exclusion of infection therefore requires a standardized diagnostic procedure with the use of algorithms shared between specialists involved in order to correctly interpret clinical and laboratory results often ambiguous.
**Materials and methods** The purpose of this study was to check the feasibility of upgrade our conventional protocol to non conventional, using systems as PCR (molecular technique), sonication and SEM (scanning electron microscope) in a standard hospital procedure. We considered 14 patients with planned revision surgery: 7considered aseptic and 7 considered septic. During the revision surgery over the standard procedure (1 sample for sinovial count, a minimum of five sample for sinovial tissue microbiology), we performed a scratch with twin scalpel blades of each component prosthesis removed. Each blade was send in microbiology and to SEM. The removed prosthesis was sent to the Microbiology Dept. for sonication on further microbiological tests.
**Results** It is a study with a small number of patients to test the feasibility of an advanced diagnostic protocol inside a hospital routine. The collected data were subjected to a critical analysis by the specialists involved in diagnosis and treatment of periprosthetic infections. We also tested an innovative line of communications for collection and data sharing between the specialists located in non-adjacent Depts.
**Discussion** The expensive molecular PCR technique in our experience does not seem to improve the diagnostic quality compared to traditional methods. Sonication requires an accurate setup in order to allow the detachment but not the destruction of the biofilms, while the SEM seems to be very sensitive.
**Conclusions** It is reasonable to believe that the infection rate is underestimated but the knowledge need a continuous acquisition, analysis and real-time sharing of information collected, in order to avoid errors of over- or underestimation. In this context our protocol seems to be reasonably feasible and SEM is a new clinical tool that deserves our future attention.

**Suggested readings**

1. Bauer Tw et al. (2001) J Bone Joint Surg Am 88:869–882
2. Zimmerli W et al. (2003) Infection 31:99–108
3. Mason JB et al. (2003) J Arthr 18:1038–1043
4. Gallo J et al. (2004) Biomed Pa. 148(2):123–129
5. Trampuz A et al. (2004) Am J Med 117:556–562.
6. Ferrari R, Castelli C (2011) Eur Cells Mat 21(Suppl 2)

### *C54–FOOT AND ANKLE 3*

### The painful prostheses in the treatment of hallux rigidus

R. Valentini*, G. Piovan, S. Bernobi, P. Dordolin, G. Fancellu

Clinica Ortopedica e Traumatologica, Università degli Studi di Trieste (Trieste, IT)

**Introduction** The degenerative joint disease of the hallux rigidus in the advanced stage has always been a challenge in surgical treatment. Over the years there have been several proposals of joint replacement surgical techniques with the aim of relieving pain, correcting deformity and maintaining a degree of motion. We first used the osteotomy proposed by Valenti and then the technique of replacing the head of the first metatarsal with a bioresorbable spacer (Giannini). With this technique we have shown consistently positive results in regard to the satisfaction of patients with increased range of motion, but with the appearance, sporadic, of an important inflammatory reaction.
**Materials and methods** In recent years, for the treatment of hallux rigid severe, we used a metal arthroplasty (Reflexion). We checked 25 patients (18 female, 7 male) of mean age 58.1 years, operated with this technique from June 2008 to June 2011. Truncal anaesthesia is performed on the ankle and is granted the immediate loading. The patients were followed up clinically and with radiographs (medium follow-up 18 months). The functionality was evaluated with the score of Kitaoka et al.
**Results** The return to normal activities was around 28 days (min 21 max 65). The mean score second Kitaoka was increased to 75 points at 3 months postoperative and 87.1 points after 6 months, starting from a preoperative score of 36.6.; the average ROM was 42° (extension and flexion 25.3°, 18.1°). We had a loosening of the implant with removal of prosthesis and the appearance of valgus of first MP with occasional pain in 4 cases.
**Discussion** Over the years various techniques have been proposed to alleviate pain, restore and maintain the motility of the first MF in patients with hallux rigidus. Our results appear to be in line with those obtained by other Authors who use prosthetic replacement. That research should go towards improving the osteointegration of prosthetic stems to prevent the mobilization and the pain, perhaps creating anatomical stems with more "fit." Only then prosthetic replacement of the first MP can be considered one of the best surgical solutions.
**Conclusions** The results seem to be favourable, as even patient satisfaction is complete. We must improve the design prosthetic to get positive results to be comparable to those of prostheses of the knee and hip. We believe, however, that with this technique it maintains a certain degree of motion of the joint guaranteeing a good feature of the forefoot.

### Infective risk control in internal fixation devices with negative pressure medications. Work in progress: a study on 8 cases

R. Mazzone*, M. Ceretti, G. Monacelli, S. Pappalardo, P. Ottolenghi

Ortopedia e Traumatologia D, Policlinico Umberto I, Università di Roma "Sapienza" (Rome, IT)

**Introduction** The internal fixation devices are indicated in emergency fractures exposure. Their exposure represents a severe bone infective risk condition. Nowadays in literature and in clinical experience the golden standard treatment for infective internal fixation devices exposure is their removal because the developing biofilm is refractory to antibiotic therapies. The last one condition compromises the surgical bone osteosynthesis stability.
**Materials and methods** We have enforced a protocol based on NPWT in internal fixation devices exposure. The crisis patients were 8 with an age between 32 and 66 years. After skin lesion closing (without internal fixation device removal) we checked patients at follow-up.
**Results** After 2 years from the first experience we have not found any infective series which was proved by X-rays, blood or clinical exams in the 8 followed cases.
**Discussion** The follow-up was 3 years after skin lesion healing. We can express our final opinion about NPWT efficacy.
**Conclusions** We will discuss the NPWT efficacy and possible alternative options.

### Acute injury of Achilles tendon: our experience with percutaneous tenorraphy (Tenolig)

L. Liguori*, D. Greco, G. Centaro, M. Sciaccaluga

(Cuneo, IT)

**Introduction** The aim of surgical treatment of Achilles tendon lesions is to ensure good healing and early mobilization, avoiding or at least reducing bad surgical outcomes. Percutaneous tenorraphy with Tenolig allows these results.
**Materials and methods** We report our experience on 103 patients (85 males and 18 females; mean age: 48.5 years—range 19–86; follow-up: 6–132 months) visited for subcutaneous rupture in middle third of Achilles tendon between 2000–2011.
**Results** AOFAS score was available in 68 patients. In all cases there was no difficulty in daily activities recovery, meanwhile return to sports was highly variable, depending from pre-injury level of activity. In no case there were limitations in mobility of tibiotarsal joint.
**Discussion** Percutaneous tenorraphy with Tenolig is indicated in all cases of acute subcutaneous rupture in the middle third of Achilles tendon, even in patients making sports. Indication can be extended to aging and diabetic patients with worsen microcirculation. There was no indication in inveterate ruptures and in miotendinous lesions.
**Conclusions** The technique is based on respecting biological healing principles, guaranteeing a stable approximation of the tendon fragments and so an early mobilization. Our decennial experience suggests that percutaneous suture with Tenolig is effective and safe for what concern unfavourable outcomes typical of standard surgery.

### Lisfranc fracture-dislocation: treatment with absorbable fixation

W. Daghino*[1], E. Bettoni[2], G. Vasario[1], G. Massazza[1], B. Battiston[1]

[1]Ospedale CTO (Turin, IT);
[2]Scuola di Specializzazione in Fisiatria, Università di Torino (Turin, IT)

**Introduction** In the surgical management of the Lisfranc fracture-dislocation we have to choose between metal screws, that will be removed after some time for preventing the risk of rupture and Kirschner's wires. This presents a high risk of correction loss.
**Materials and methods** The patients diagnosed for a Lisfranc fracture-dislocation and treated surgically, at the Department of Traumatology of the Orthopaedic and Trauma Center (CTO) in Turin, from January 2003 to June 2011, were re-evaluated. The patients were divided into two groups according to the type of stabilization: in the first group they were treated with Kirschner's wires only, in the other group they were treated with absorbable screw and rods, in association with metal wires or isolated, then a retrospective analysis was performed. The clinical evolution was rated with AOFAS rating scores and the radiological evolution through seriate radiography.
**Results** 26 patients were treated, 12 using Kirschner's wires and 14 using poli-L-lactic acid (PLLA) screws and rods, isolated or in association with metal wires. Analysis of data was completed for 19 patients: 9 treated with Kirschner's wires only, 10 treated also with PLLA material. The mean clinical score was higher in PLLA group, but the difference isn't statistically significant. At the radiographic control, in the Kirschner group, 3 cases of loss of correction were identified, none in PLLA group. No cases of PLLA intolerance were detected.
**Discussion** Lisfranc fracture-dislocations are rare events, often with a complicated diagnosis and with disputes related to the type of surgical stabilization. The relative small number of cases does not allow giving statistical significance to the best clinical evolution of the PLLA group, but the absence of correction loss, over the time, in this group, is an important element to be considered.
**Conclusions** The use of absorbable material for Lisfranc fracture-dislocation's stabilization seems to be a valid alternative to restrict the typical problems of this surgery; however further studies, numerically larger, are required to confirm this evidence.

### Percutaneous bunion correction: preliminary report

F. Forconi*[1], L. Miccichè*[2], V. Sessa[1], M.A. Rosa[2]

[1]U.O.C. Ortopedia e Traumatologia, San Giovanni Calibita-Fatebenefratelli (Rome, IT);
[2]Scuola di Specializzazione in Ortopedia e Traumatologia, Sezione di Ortopedia e Traumatologia, Dipartimento delle Specialità Chirurgiche, Università degli Studi di Messina (Messina, IT)

**Introduction** Over the last years, percutaneous and less invasive surgery for hallux valgus correction gained particular interest among orthopaedic surgeons and patients as well. Not so many papers on this issue are present in literature, nevertheless the latter is still debated in foot surgery congresses and meetings. The aim of our study was to evaluate preliminary results assuming indication limits on mild deformities.
**Materials and methods** From October 2010 to October 2011, 20 patients were consecutively operated on for percutaneous mild hallux valgus correction by the same surgeon. Percutaneous exostosectomy and an Akin phalangeal osteotomy were performed in all patients. Percutaneous lateral release was associated in cases with residual deformity. Radiographic pre-operative measurements were always registered under the value of 11° about intermetatarsal (I.M.) angle, the value of 30° about the hallux valgus (H.V.) angle, distal metatarsal articular angle (DMAA) was under 10° and sesamoids luxation was limited at a grade 2. Radiographic measurements were obtained in the first day, at 30 days and at 6 months after surgery. Clinical parameters were registered using the AOFAS Hallux Metatarsophalangeal Interphalangeal scale in pre and post-op period.
**Results** The mean follow-up was 10 months (4–16). All radiological measurements at follow-up were registered in a physiological range. Clinical scoring improved in any cases with good patient satisfaction.
**Discussion** Limited surgery exposure, short operative time and a limited post-operative pain are all great advantages in this procedure.
**Conclusions** Percutaneous bunion surgery demonstrated to be a reproducible and predictable technique in our study. We think it is the choice procedure in the radiological parameters we assumed, or rather in all the cases were no metatarsal translation has needed.

### *C55–TRAUMATOLOGY 8*

### "A minima" elastic synthesis with MIROS technique

G. Topa*[1], P. Ditto[2], S. Barreca[l], G. Caruso[2], M.A. Rosa[2]

[1]UO di Ortopedia e Traumatologia, Azienda Ospedaliera Bianchi-Melacrino-Morelli (Reggio Calabria, IT);
[2]Scuola di Specializzazione in Ortopedia e Traumatologia, Sezione di Ortopedia e Traumatologia, Dipartimento Specialità Chirurgiche, Università degli Studi di Messina (Messina, IT)

**Introduction** The synthesis "a minima" has always been a complementary support to the most appropriate stable synthesis which is always the primary goal. The use of this approach is a common heritage of all orthopaedic surgery in those situations where the patients have precarious clinical and/or local condition.
**Materials and methods** The MIROS (Minimally Invasive Reduction and Osteosynthesis System) is an easy and intuitive system for osteosynthesis and it is adaptable to multiple types of fractures. It consists of metal wires to be inserted into the medullary canal, or in the bone tissue for the internal fixation, and one or more "clips" for the external stabilization in the case of a synthesis of the "hybrid type".
**Results** We evaluated the results of 29 cases treated at the Ospedali Riuniti "Bianchi-Melacrinò-Morelli" of Reggio Calabria in the period between September and December 2011. These 29 cases consist of: 3 calcaneus fractures, 5 humeral fractures, 1 peri-prosthetic fracture, 3 femoral fractures in growth subjects and 17 fractures of distal metaepiphysis of the radius. X-ray controls were performed in standard projections for the different body segments, either after surgery as at 1 and 3 months.
**Discussion** MIROS system was used, as well as in its classical form and in summary "delta" synthesis which adds to the known advantages of the elastic synthesis, a greater resistance to loads to torsion and compression forces; it guarantees either the respect of vascularization thanks to the mini-invasiveness, as the respect of growth metaepiphyseal cartilage .
**Conclusions** In our study, we used the MIROS system, which we believe to be a mechanical evolution of the simple K-wire; in those instances where we used the "delta" solution we have faced surgical/clinical solutions otherwise difficult to solve. However, we believe that in its classic form this method can be used as a first approach in fractures of the distal radial metaepiphysis.

### Minimally invasive surgical techniques in the treatment of proximal femoral fractures: PCCP plate versus ITST nail. A prospective randomized matched study

E. Raimondo*[1], L. Marchesi[2], A. Pelis[2], B. Grisone [1], A. Giubilato[1], L. Pietrogrande[3]

[1]Unità Operativa Ortopedia e Traumatologia, AO San Paolo (Milan, IT);
[2]Università degli Studi di Milano (Milan, IT);
[3]AO San Paolo, Università degli Studi di Milano (Milan, IT)

**Introduction** The incidence of proximal femoral fracture is constantly increasing and is associated to an augmented surgical risk due to the age of patients and comorbidity. It is therefore desirable to reduce surgical complications using minimally invasive surgical techniques such as PerCoutaneous Compression Plate (PCCP) and traditional nailing (TN) techniques. Aim of the study was to compare the functional ability of the elderly patient after these two different surgical techniques.
**Materials and methods** A 12-month randomized matched study was designed. Seventy patients (ten men and sixty women; age range 48–98 years) were recruited between 2006 and 2010 at our Unit (35 PCCP, 35 ITST) and matched for age (± 4 years), sex, type of fracture (according to AO and EVANS indexes), comorbidity (evaluated with ASA and Charlson Index), duration of preoperative hospitalization and type of anaesthesia (general or locoregional). Duration of intervention, blood transfusion, post-operative hospital stay were recorded and complications monitored during the study. Functional recovery of patients was evaluated before and at 40 days, 6 and 12 months after surgery by the Modified Harris Hip Score.
**Results** Rate of post-surgery blood transfusion was lower in PCCP than ITST (68.6 vs. 97.1 %; $p < 0.001$). Infection occurred in one PCCP patient and in one ITST patient. One patient in ITST group showed proximal screw loosening. Six patients (8.6 %) died during the study period (2 PCCP, 4 ITST; $p = 0.673$). Mean (SD) pre-intervention HHS was 73.6 (10.3) and 73 (10.4) in PCCP and ITST, respectively ($p = 0.809$). At 40 days after surgery PCCP group exhibited higher mean (SD) HHs value than ITST group [55.3 (11.2) vs. 50 (10.5); $p < 0.05$], while no significant difference between groups was found at 6 [64.4 (10.1) vs. 61.9 (9.2)] and 12 [72.1 (10.8) vs. 68 (9,2) months.
**Discussion** Both these surgical techniques seem to be easy to implant, minimally invasive and with a low rate of surgical complications. Therefore PCCP patients seem to need less blood transfusions probably due to a minor impact on bone marrow. Functional recovery based on HHS was faster in PCCP patients.
**Conclusions** On our data PCCP seems to allow less blood transfusions and faster functional recovery and show a superior minimal invasiveness.

### Femoral neck fracture in an extremely rare case of oncogenic osteomalacia: case report

C. Ruosi*[1], A. De Caro[1], S. Liccardo[1], A. Barbato[1], G. Colella[1], S. Lupoli[1], D. Rendina[l], F. Galletti[2]

[1]Dipartimento di Ortopedia e Traumatologia, Università degli Studi di Napoli "Federico II" (Naples, IT);
[2]Dipartimento di Medicina Clinica e Sperimentale, Università degli Studi di Napoli "Federico II" (Naples, IT)

**Introduction** Oncogenic osteomalacia is a rare paraneoplastic syndrome caused by an increase in FGF23 (slightly more than 100 cases reported worldwide). FG23 is a recently identified hormone which regulates D vitamin's and phosphate's levels, it is usually secreted by benign mesenchymal tumors, small in size and, therefore, difficult to identify. In most of the cases cancer is located at the level of long bones or massive facial. The symptomatology, frequently unspecific, is characterized by weakness, worsening myalgia and bone pain, especially in areas under the load. At bone it is observed an increased deposition of a not sufficiently mineralized bone matrix with consequent reduction of the resistance to mechanical load and greater risk of pathologic fractures.
**Materials and methods** A 76 year-old patient with severe diffuse demineralization, many vertebral hemangiomas, who was treated for about 10 years with replacement therapy. Hospitalized at our department due to a basi-cervical fracture to the left femur following an accidental fall, underwent to surgical reduction and synthesis with intramedullary nail locked to the left femur bone. In the perioperative period, the complete absence of cancellous trabeculae in the proximal femur's metaphysis caused enormous difficulties. The prediction of delayed union and poor bone density assessed intraoperatively, led us to practice pulsed electromagnetic fields in combination with active and passive mobilization and absolute prohibition of loading for 3 weeks of the left hip.
**Results** Post-surgical therapy with PEMF of the left hip for 3 weeks, with the absolute prohibition of loading and mobilization alone A/P of the left lower limb. Clinical and radiographic control after 3 weeks, showed a good nail placement without signs of screws' mobilization. After 3 weeks he started a protected loading with walker and only after 5 weeks passed to the use of double stick. Walking without any support 2-month after surgery.
**Discussion** Oncogenic osteomalacia is often associated with bone and soft tissue cancerous lesions that cause hypophosphatemia by releasing circulating factors, commonly called phosphatonine. As demonstrated in literature, a partial or total resection of the primary tumour often leads to persistent hyperphosphaturic hypophosphataemia, which leads to the need to continue drug therapy and the use of additional treatments to promote the formation of bone callus.
**Conclusions** Early application of PEMF allowed a rapid functional recovery despite severe demineralization and delayed bone union. However, the osteogenetic stimulation must be implemented only when the stability, alignment, and the contact between the fracture's bone ends are guaranteed.

### Lisfranc lesions: what treatment options?

A. Formica[1], S. Pelle*[2], L. Magistro[1], A. Mattei[1], A. Impagliazzo[3]

[1]Azienda Ospedaliera San Giovanni Addolorata (Rome, IT);
[2]Università degli Studi di Roma "Sapienza" (Rome, IT);
[3]Casa di Cura Addominale EUR (Rome, IT)

**Introduction** Tarso-metatarsal joint injuries are often characterized by diagnostic and therapeutic difficulties, and long-term residual functional limitations and disabilities. Several treatment options were purposed but scientific production still provides no clear data.
**Materials and methods** The objective of this study was to compare our experience results with other studies data available in the literature, in order to define a suitable treatment protocol for Lisfranc injuries. We performed a retrospective study of patients with Lisfranc injury; politrauma, and patients with concomitant homolateral lower limb lesions or open fractures, were not included. The population includes 25 patients, 18 male and 7 female (age 32–64 years) with monolateral Lisfranc lesion detected by hind-foot X-rays and/or CT scan. Treatments applied were: (1) closed reduction and cast immobilization (n = 2); (2) closed reduction and percutaneous stabilization by K-wire or screws (n = 7); (3) open reduction (with dorsal approach centred on first intermetatarsal and fourth intermetatarsal space, if required) and synthesis with screws (n = 16); (4) no patient was treated with primary arthrodesis. All patients were subjected to clinical examination (pain, gait impairment, orthoses need, functional outcome), X-rays and baropodometry (static and dynamic evaluations) (follow-up 5–10 years). We employed AOFAS Ankle-Hindfoot Scale in order to compare our results to international studies outcomes.
**Results** Our results support the concept that stable anatomical reduction of fracture-dislocations of the Lisfranc joint leads to the best long-term outcomes; closed reduction and K-wire or screw percutaneous fixation are suitable only in special cases, such as trauma-related vascular or cutaneous problems. Primary arthrodesis is recommended for highly complex lesions.
**Conclusions** Open reduction and internal fixation is recommended as the treatment for most injuries of the Lisfranc joint: anatomical reduction remains important for a good long term outcome. Tarsometatarsal arthrodesis should be considered as salvage procedure in case of painful posttraumatic arthritis with significant functional consequences.

### *C56–PAINFUL PROSTHESES 6*

### Thigh pain and short stems: new implants or usual problems?

F. Leonardi*, F. Rivera

Dipartimento di Ortopedia e Traumatologia, Ospedale SS Annunziata, ASL CN1 (Savigliano, Cuneo, IT)

**Introduction** Short stems, or metaphyseal stems, are designed and introduced in orthopaedic surgery to get better results versus cementless traditional stems.
**Materials and methods** From January 2010 to September 2011 in our Hospital, 184 short stems in 178 patients were implanted. All patients were recorded pre-op. and follow-up was performed at 1, 3, 6 months and 1 year with clinical (HHS, Womac) and radiographic evaluation.
**Results** The retrospective analysis identified 2 groups of patients with unilateral total arthroplasty and coxarthrithis diagnosis. The first group, 62 patients (56 operated with anterior approach, 44 with posterior approach), with a short straight stem; the second group, 104 patients (58 operated with anterior approach, 44 with posterior approach) with short anatomic stem. We also had a third group of patients, operated between March 1996 and September 1998 using a short anatomic stem with neck preservation (182 patients, posterior approach). No statistically difference was found comparing the clinical results of the three groups ($p > 0.1$), although follow-up differences between groups 1 and 2 and group 3. Complications analysis gave us different results. Actually we had 4 subsidence cases (6 %) after 3 months from operation in group 1, two of which were revision cases for pain. In group 1 we had two cases of subsidence (2 %) after 3 months without ulterior consequences about implant stability. We had 1 intra-operative neck fissure in group 1 and, in group 2, 2 intra-operative neck fissure and 1 intra-operative proximal femur epiphysis fracture with revision of implant after 2 days.
**Discussion** We need a longer follow-up to confirm good results published in literature about short stems use in short-medium-terms. Those results are however published by the designers of the stems.
**Conclusions** Aim of our study is the need to have a short stem, preferably an anatomic one, with no excessive pro-bending, to obtain high mechanical interferences and, at the same time, not to have intraop. fractures, with neck preservation to avoid instability in rotation, and finally with a surface treatment to increase primary stability in precocius phases.

### Algorithm for the pre-operative diagnosis of knee implant infection

D. Tigani*[1], L. Amendola[2], L. Savarino[3], N. Baldini[3], V. Bochicchio[4]

[1]Ospedale Santa Maria alle Scotte (Siena, IT);
[2]Ospedale Maggiore (Bologna, IT);
[3]Istituto Ortopedico Rizzoli (Bologna, IT);
[4]Ospedale S. Orsola-Malpighi (Bologna, IT)

**Introduction** Deep infection occurring in a total knee arthroplasty is one of the most devastating complications. Early diagnosis may improve outcomes allowing appropriate surgical planning and early antimicrobial treatment.
**Materials and methods** Thirty-one consecutive patients with total knee arthroplasty failure were enrolled to assess our algorithm, analyzing the clinical, radiographic and laboratory parameters used. A follow-up of 2 years was considered to validate our pre-operative algorithm.
**Results** No test was found to be 100 % sensitive and specific. TC-99 scintigraphy was positive in 29 cases of loosening out of 31. Granulocyte scintigraphy was positive in all cases with sepsis, except for one underwent a prior antibiotic therapy; in all aseptic cases it was negative. Leukocyte number did not show any significant difference. On the contrary ESR, CRP and fibrinogen significantly increased and the optimal infection diagnosis cutoffs were determined. Synovial fluid culture resulted in 2 false-negative in septic cases and 2 false-positive in aseptic cases.

**Discussion** We believe that, after an accurate clinical and radiographic evaluation and in the presence of a positive TC-99 scintigraphy, a screening test including three inflammation parameters is advisable. An elevation (higher than the cut-off) of at least two parameters should prompt further investigation to include or exclude infection. With positive inflammation tests and granulocyte scintigraphy, we always proceed with the needle-aspirate culture, for the germ isolation and the antibiotic resistance evaluation. With positive serological tests but negative granulocyte scintigraphy, culture and leukocyte count on needle-aspirate are suggested to diagnose infection. If needle-aspirate culture does not agree with leukocyte count, a new aspirate after 3 weeks is performed and inflammatory markers are repeated. If all the tests are negative, a CT scan is useful to detect a malrotation, whereas reflex sympathetic dystrophy or neuroma around the knee can be suspected in the presence of a negative CT scan. If granulocyte scintigraphy and sierological tests disagree, a second needle-aspirate should be considered. In case of positive test, a two-stage exchange is advisable. Intra-operative biopsies at the prosthesis or spacer removal are needed in order to confirm or exclude by histomorphometry the infection persistence.
**Conclusions** Adherence to this algorithm could contribute to preoperatively define a rational surgical and antibiotic treatment strategy. The major limitation of the study was the low number of subjects.

### Surgical revision in knee joint prosthesis

F. Martire*, A. Crescibene, G. Gentile, S. Anastasio, S. Grande, M. Candela

U.O.C. di Ortopedia e Traumatologia (Paola, Cosenza, IT)

**Introduction** Failure of knee joint prosthesis due to various causes such as septic mobilization, infection, articular instability, prosthetic material wear, articular rigidity and improper patient selection. Pain following surgery is the main reason for prosthetic revision, in almost all cases the origin of pain is evident but in some cases a painful prosthesis is without apparent cause.
**Materials and methods** 102 knee joint prosthetic surgical replacements were performed between January 2007 and December 2009 at the Orthopaedic and Traumatology Unit, Paola Hospital. 7 prostheses were cut, 5 female e 2 male subjects, causes were 2 septic mobilization, 3 cases of sepsis, painful prosthesis in 2 cases; I.O.C.T evaluation score was used along with serial follow-up radiographs.
**Results** Clinical data analysis resulted in statistically significant improvement of clinic parameters of the revised knee joint prosthesis, we can state that revision replacement of knee joint prosthesis in most cases results in good functional and clinical recovery of the joint although surgery is not free from intra-operative risks and post-operative complications.
**Discussion** The main goal of revision of painful knee joint replacement prosthesis lies in the elimination of the pain symptomatology, functional recovery and return to daily life activity. Bone tissue quality, eventual surgery complications and patient compliance determine, ultimately, the possibility of implanting a new prosthesis.
**Conclusions** Painful prosthesis is a difficult subject to understand and resolve that is due to multifactor determinants; in many cased the aetiology remains unknown; serial clinical and instrumental follow-up is preferred to prompt prosthetic revision that exposes the patient to new surgery risks and reduces patient satisfaction.

### Pain assessment in TKA

F. Atzori*[1], L. Sabatini[1], A. Massè[1], A. D'amelio[2]

[1]S.C.D.U. Ortopedia e Traumatologia, A.O. Universitaria San Luigi Gonzaga di Orbassano (Orbassano, Turin, IT);
[2]Scuola di Specializzazione in Ortopedia e Traumatologia, Università degli Studi di Torino (Turin, IT)

**Introduction** The results of total knee replacement in patients with knee osteoarthritis are good, improving pain relief, joint function and quality of life. However, some patients report pain even after the implantation of a knee prosthesis, and sometimes for unclear reasons; in these cases, the pain is frequently localized in the front area of the knee.
**Materials and methods** Through a comparison of our series (approximately 100 cemented implants and revisions of three different models) with the data in the literature, the objective of our work is to obtain an overview of pain after prosthesis knee; we debate the possible reasons for the onset of pain, the reasons that lead to a knee revision and a possible connection between pain and different concepts of prosthetic designs.
**Results** Our data agree with what is stated in the literature: the main causes of pain after implantation of knee prostheses are aseptic loosening, infection, polyethylene wear, arthrofibrosis and patellar problems.
**Discussion** We should identify the cause of pain after total knee arthroplasty, and this is through a medical history (do not forget to evaluate medical problems such as depressive syndromes), a precise examination, and a accurate evaluation of imaging. Only the precise identification of the cause of pain may provide the most appropriate pain management.
**Conclusions** It is difficult to establish the optimal treatment of postoperative pain in a patient with total knee arthroplasty, also for the lack of meta-analysis or high quality randomized trials; we need a flow chart making it possible a correct pain score and pain aetiology evaluation.

### *C57–PAEDIATRIC ORTHOPAEDICS 2*

### Gait analysis as a method for monitoring hemophilic patients

L. Marengo*[1], A. Andreacchio[2], E. Forneris[3], M. Messina[4]

[1]I Clinica Ortopedica, Università degli Studi di Torino (Turin, IT);
[2]S.C. Ortopedia e Traumatologia, Azienda Ospedaliera OIRM Sant'Anna (Turin, IT);
[3]Servizio di Riabilitazione, S.C. Ortopedia e Traumatologia, Azienda Ospedaliera OIRM Sant'Anna (Turin, IT);
[4]S.C. Immunoematologia e Medicina Trasfusionale, Azienda Ospedaliera OIRM Sant'Anna (Turin, IT)

**Introduction** Haemophilic arthropathy is one of the most serious haemophilia complications and it is the consequence of recurrent hemarthrosis. Repeated bleeding episodes induce synovitis that is irreversible and may progress resulting in an impairing functional limitation and it compromises individual's autonomy. Gait analysis (the systematic study of human walking) allows for the detection of the minimal gait disorders and earlier diagnoses and rehabilitative treatment to be made, to prevent or minimize functional limitations.

**Materials and methods** 53 children affected by haemophilia were identified. They had ranged in age from 0 to 18 years. Gait analysis was only performed on patients aged from 4 to 18 years: 47 patients underwent gait analysis tests, the average age was 11.4 years. The duration time of study was 2 years.
**Results** Posture and disturbance disorders are more serious in patients affected by haemophilic arthropathy rather than in haemophilic patients who don't suffer from arthropathy. Postural assessments and gait analysis show kinematic and kinetic parameters are significantly different than healthy people. Speed and step length decrease while double support and swing phase increase.
**Discussion** At the end of the first year of study, analysis and comparison of postural assessments and gait analysis tests show that all kinematic and kinetic parameters are altered in all patients suffering from haemophilic arthropathy. Gait analysis parameters change even in children who experienced just one bleeding episode. A single episode of hemarthrosis can damage intrarticular structures resulting in changes of posture and gait pattern, according to Young Hudacek's studies (1954).
**Conclusions** According to Dormans et others from Philadelphia we started using the same therapeutic approach. One point of strength is the economical cost of treatment which is cheaper compared with others surgical techniques. On the other hand the Scaglietti's method needs several surgeries, which it means several anaesthesias.

### Femoral shaft fractures in children: nonoperative treatment versus external fixation

I. Sanzarello, E. Calamoneri, S. Mauro, L. D'andrea, M.A. Rosa*

Scuola di Specializzazione in Ortopedia e Traumatologia, Sezione di Ortopedia, Dipartimento Specialità Chirurgiche e Traumatologia, Università degli Studi di Messina (Messina, IT)

**Introduction** Paediatric femoral diaphyseal fractures are commonly related to a good prognosis. Several methods of treatment are displayed in Literature (elastic stable intramedullary nailing, external fixation, plate osteosynthesis). Non-operative treatment is the gold standard in patients younger than 6 years because of the excellent properties of repair and remodelling of the bone. Transkeletal or tanscutaneal traction followed by hip spica casting is, nowadays, less accepted because of the lengthening of hospitalization and the higher discomfort of the patient.
**Materials and methods** The study involved 13 patients, 8 males and 5 females aged between 2 days and 8 years (mean age 3 years and 7 months), and divided into two groups based on: type of fracture, age, grade and stability of the achieved reduction. In group 1, consisting of 6 patients, treatment consisted of immediate reduction under anaesthesia with early hip spica casting; in group 2, consisting of 7 patients, reduction under anaesthesia and axial external fixation was performed. The results were evaluated on different parameters (ROM, dysmetria, defects and/or delayed consolidation) with a mean clinical and radiographic follow-up of 3 years and 6 months.
**Results** Clinical results were nearly the same between the two groups. None of the patients had pain, muscle strength was normal in 80 % of cases, good in 20 % (MRC scale); functional analysis of the movements showed a complete recovery of the joint ROM. Three years follow-up showed a mean dysmetria, secondary to overgrowth phenomenon, less than or equal to 0.3 cm in group 1 and less than or equal to 0.5 cm in group 2. There was no clinically significant limping, neurological and/or associated vascular disorders or any other kind of late complications. There were also no reports of nonunion.
**Discussion** Treatment choice depends from several factors: type of fracture, degree of decomposition and/or comminution, general condition, age and patient's comfort. Non-operative treatment, where possible, it is commonly accepted as the gold standard in patients younger than 4 years.
**Conclusions** Our pick of external fixation as the peculiar operative treatment is influenced by our mastery of this technique maturated in adult traumatology, and, in our experience, is extremely reliable despite the restricted indications assigned from the Literature, ensuring good results with reduced complications.

### Calcium sulphate pellets for treatment of unicameral bone cysts in children

A. Andreacchio*[1], L. Marengo[2]

[1]Ospedale Infantile Regina Margherita (Turin, IT);
[2]II Clinica Universitaria, Azienda Ospedaliera CTO/Maria Adelaide (Turin, IT)

**Introduction** Unicameral bone cyst (UBC) is a common benign tumor which induces a progressive bone weakness of the involved bone, often resulting in a pathological fracture. More than 50 % of the UBCs is discovered because of this acute event.
**Materials and methods** The technique, which is purposed in this study, is a minimally invasive procedure: a 3 cm long skin incision is made over the midpoint of the cyst, a small fenestration is created in cortical bone, curettage of the litic lesion and then medullary canal is opened. Finally, sulphate pellets are deployed in order to completely fill the cyst. During this last phase, fluoroscopic control has a paramount issue. When complete filling of the cyst is accomplished, small wound can be closed. In the post-operative period brace arm, no cast is required. This surgical technique, described by Dormans et al. (Journal of Paediatric Orthopaedics 2005), was performed to treat 12 unicameral bone cysts in 12 patients. The involved bones were humerus (11 cases) and distale radius (1 case). Patient had ranged in age from 4 to 18 years. Radiographs were taken at postoperative day 1 and 1, 3, 6 and 12 months after surgery. Follow-up period ranged from 6 to 18 months.
**Results** Results were evaluated on plain radiographs according to the classification system of Capanna et al. 10 UBC were classified as completely healed and 2 as healed with residual radiolucency. One month after surgery, radiographs showed complete resorption of the sulphate pellets. Cortical thickening was observed in all patients and none of them experienced further pathological fractures.
**Discussion** In published orthopaedics literature, many surgical techniques are proposed to treat this benign bone tumour. Upper limb has lower risk of complicated pathological fracture compared with the lower limb. The ideal treatment should be less invasive as possible but effective.
**Conclusions** According to Dormans et others from Philadelphia we started using the same therapeutic approach. One point of strength is the economical cost of treatment which is cheaper compared with others surgical techniques. On the other hand the Scaglietti's method needs several surgeries, which it means several anaesthesias.

### Flat foot of the infancy dealt with endo-orthotic implant calcaneal stop type: our experience

P. Barbato*, W. Leonardi

Arnas Garibaldi (Catania, IT)

**Introduction** The flat foot can have surgical indication in patients between the 8 and 14 years, we prefer the interval of age between the

10 and 12 years. The objective is to restore the relationships of the coxa pedis. We describe turns out obtained with the use of arthorisis by means of endo-orthotic implant calcaneal stop type.

**Materials and methods** From June 2005 to December 2011 we dealt 183 patients, 67 male and 116 female, and 361 feet with the surgical technique of the calcaneal stop. In 5 cases the medial plastic was associated. The comprised age was between 10 and 14 years with a maximum follow-up of 6 years.

**Results** Patients were evaluated during follow-up with clinical examination and podoscope, with AOFAS score and X-rays (Meary's line, calcaneal pitch, lateral talocalcaneal angle, talonavicular coverage angle, Kite's angle). The axis of the back foot improved in 94.2 %, in 2 cases it was necessary to remove the endo-orthotic implant for intolerance. Medium score AOFAS is passed from 80.57 to 98.21 points and the 87.81 to the 98.60. Also Meary's line and talonavicular coverage angle improved.

**Discussion** The percentage of satisfied patients turned out to be of 91.3, 87.6 % of the patients returned to practice sport activity without pain. The progressive cargo was granted to 2 days after surgery; in the 5 cases in whom medial plastic was executed one the patient it is remained with chalk to ankle boot for 4 weeks. In these cases the complete resumption of sport activity happened after approximately 60 days from surgery; in the remaining cases immobilization was not prescribed.

**Conclusions** The correction of the juvenile flat foot with endo-orthotic implant therefore turned out satisfactory. It is in immediate post the operating one, at a distance of time with remission of the pain and it allows fast sport resumption in an elevated percentage of cases. Moreover, it is a simple and reliable surgical technique, however it demands a correct diagnostic organization for a corrected surgical treatment.
